# Depressive and anxiety disorders in Polish children across the COVID-19 pandemic: a nationwide registry-based time-trend analysis

**DOI:** 10.3389/fpsyt.2025.1728383

**Published:** 2026-01-06

**Authors:** Marcin Czech, Malwina Hołownia-Voloskova, Katarzyna Bliźniewska-Kowalska, Krzysztof Marcin Zakrzewski, Otton Roubinek, Anna Mosiołek, Andrzej Silczuk

**Affiliations:** 1 Department of Pharmacoeconomics, Institute of Mother and Child, Warsaw, Poland; 2 Department of Adult Psychiatry, Medical University of Lodz, Lodz, Poland; 3 The Independent Group of Public Ambulatory Care Institutions Warsaw-Ochota, Warsaw, Poland; 4 Pharmacy and Biotechnology Center, Lukasiewicz Research Network - Industrial Chemistry Institute, Warsaw, Poland; 5 Department of Interdisciplinary Disability Studies, The Maria Grzegorzewska University of Special Education, Warsaw, Poland; 6 Department of Community Psychiatry, Faculty of Life Sciences, Medical University of Warsaw, Warsaw, Poland

**Keywords:** anxiety disorders, children and adolescents, COVID-19, depression, mental health, poland, psychotropic drugs, public health

## Abstract

**Introduction:**

The COVID-19 pandemic profoundly disrupted the mental health landscape for children and adolescents in Poland. This study presents a comprehensive analysis of psychotropic medication use, sick-leave days, and psychiatric treatment rates among Polish youth from 2018 to 2024, encompassing pre-pandemic, pandemic, and post-pandemic periods.

**Methods:**

In the study we used national-level datasets. Data on Rx (recipe) purchases were obtained from IQVIA Pharmascope database for medication consumption by patients in community pharmacies. We have further examined the combined annual number of sick-leave days for 2020–2024. This data was obtained directly from the Social Insurance Institution of Poland. Lastly, we considered the dataset consisting of annual counts of children, who received at least one intervention in psychiatric care during the examined period.

**Results:**

We observed a sharp increase in antidepressant, anxiolytic, and antipsychotic prescriptions during the pandemic peak in 2021, followed by divergent post-pandemic trends—short-acting anxiolytics declined, while antidepressants and antipsychotics remained elevated. Only sertraline shows no clear peak, but rather a steady increase over the analyzed period. Concurrently, sick-leave days due to mental health diagnoses and the number of treated patients rose significantly, indicating sustained psychological distress beyond the acute crisis. Statistical analysis revealed strong upward trends for specific medications, such as escitalopram and quetiapine, and highlighted the pandemic’s distorting effect on prescribing patterns.

**Discussion:**

These findings underscore the long-term impact of COVID-19 on youth mental health and emphasize the need for enhanced early intervention, expanded access to psychiatric care, and robust public health strategies tailored to children and adolescents.

## 1 Introduction

The COVID-19 pandemic struck world-wide at the end of 2019 and seriously affected Europe from the beginning of 2020, introducing an unparalleled public health emergency with important collateral effects at the social, economic and psychological levels. Like in many other countries, the pandemic upended almost every aspect of life in Poland, and children and young people bore the brunt of it (1, 2).

In late 2019 a novel coronavirus (SARS-CoV-2) was diagnosed in Wuhan, China, and by the close of 2022, > 660 million had been infected with and > 6.7 million had died of the disease worldwide. After the first SARS-CoV-2 case was confirmed on 4 March 2020, cases rose up to the hundreds by the middle of March where the Prime Minister announced the state of epidemic on 20 March 2020 (3). Days before the declaration, the government closed schools and universities from 11 March onwards and reintroduced border controls from 15 March in an attempt to stop the spread of the virus (4).

It is now well established that periods of major crisis and upheaval consistently act as catalysts for the rise of mental illness, and there are already emerging signs of this among young people in COVID-19. Many multinational studies have observed that levels of depressive and anxiety symptoms in children and teenagers roughly doubled, compared with pre-pandemic levels, and that one in four young people reported clinically significant depression, while one in five reported anxiety symptoms during the first year of the pandemic, according to a meta-analysis (5). In Poland, in particular, it was the country´s adolescents who turned out to be particularly susceptible: studies in (and after) the pandemic report continually higher incidences of both depression and anxiety, and they are among the most frequently noted types of pandemic-related mental health disturbances in this age group (6).

The clinical struggles have played out amid limited mental health resources in Poland’s publicly funded system. In 2023, the total health care spending was 241.6 billion PLN (7.1% of GDP), but only 3.7% of the total budget have spent for mental health care (7, 8).

Human resource deficiencies amplify financial restrictions: Only 543 child and adolescent psychiatrists were practicing in Poland by April 2023, or 9.57 specialists per 100–000 underage citizens, whereas the number of psychiatrists at large settled at 9 per 100–000 population (8, 9).

Given such systemic constraints, there is an urgent need to enhance the quality of early-intervention services and to improve access to evidence-based treatments like psychosocial and psychopharmacological interventions among Polish youth.

Before COVID-19, Poland was already observing a growing epidemic of mood disorders among children and adolescents. From 2005 to 2016, the percentage of all patients under age 21 treated for major depression increased from 1.9% to 4.1%, the study found. Despite this increasing prevalence, services for another mental health were under-resourced with poor availability of specialist care, especially in the rural areas. The continued stigma associated with mental health problems also delayed diagnosis and treatment (10). The COVID-19 pandemic brought novel difficulties to children and adolescents. Lockdowns and school closures, social isolation and family stress led to disrupted daily routines and support. Research conducted in this period showed a growing number of mental disorders among the Polish youth. There were also more children reporting suicidal thoughts in the early months of the pandemic in 2020, indicating that the crisis has taken a heavy toll on youth mental health (11).

Mental health problems among children and adolescents are becoming an increasingly pressing public health issue worldwide. The World Health Organization reports that one in five children and adolescents suffer from mental or behavioral disorders, and suicide is the fourth leading cause of death among teenagers globally (12). According to recent epidemiological data by Kieling et al. (2024), the prevalence of mental disorders is 6.80% in the 5–9 age group, 12.40% in the 10–14 age group, 13.96% in the 15–19 age group, and 13.63% in the 20–24 age group (13). More than one-third of mental health issues begin before the age of 14; however, many of these cases remain undiagnosed and untreated, exerting a significant negative impact on the young individual and their family, as well as on communities, civil society, and the economy (14). Early intervention and preventive strategies are crucial, as mental disorders emerging at a young age often persist in adulthood, affecting education, employment, and overall quality of life (15).

**Figure 1:**
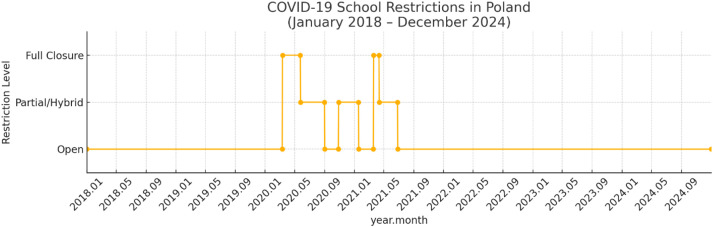
Timeline depicting the levels of COVID-19–related school restrictions in Poland from March 2020 through June 2021 Legend: Open: full in-person teaching; Partial/Hybrid: limited in-person (e.g., only youngest grades, or mixed remote/in-person); Full Closure: all-remote instruction.

Key transitions reflect the initial nation-wide closure in March 2020, phased partial reopenings in late May and September 2020, hybrid periods in autumn and winter, and the spring 2021 lockdown followed by gradual return to full in-person schooling as shown on **Figure 1** (16, 17). It is thus crucial to examine depressive disorders in Polish children and adolescents before, during, and after the COVID-19 pandemic to fully appreciate the range and consequences of this worldwide crisis on the mental health of young people. The pandemic has introduced a cacophony of stressors, social isolation, disrupted school, shrinking access to care and heightened familial tensions that have been closely associated with surges in psychological distress among children and adolescents. Before the pandemic, depression in the Polish population stood at a high level among young people with the huge treatment gap, low access to mental health care system and strong stigma. The COVID-19 pandemic exacerbated these difficulties, adding further burden to already overwhelmed provisions services and exposing the structural shortcomings of the support systems. The main goal is to describe throughput of psychotropic medications for Polish children upon the passage of time, in particular it is to estimate and compare the quantity of dispensed antidepressant, anxiolytic and antipsychotic packages during pre-COVID (2018–2019), COVID pandemic (2020–2021) and post-COVID (2022–2024) years.

The primary aim of this study was to characterize how psychotropic medication use in Polish children changed over time specifically, to quantify and compare the volume of antidepressant, anxiolytic, and antipsychotic dispensed DOT in the pre-COVID (2018–2019), pandemic (2020–2021), and post-COVID (2022–2024) periods. By mapping these dispensing patterns, the study seeks to illuminate the pandemic’s impact on pediatric mental-health treatment in Poland.

Such a study holds significant value for public health planning, educational policy, and social services. It also contributes to the broader international discourse on the long-term mental health implications of the pandemic, providing Poland-specific insights that can guide future policy and investment. Moreover, generating and disseminating evidence on the mental health of children can help to reduce stigma, encourage early diagnosis, and foster a more supportive environment for youth experiencing depression.

## 2 Materials and methods

We examined use of antidepressants and antianxiety medications among the population. Data on Rx purchases were obtained from IQVIA Pharmascope database for medication consumption by patients in community pharmacies. Used units were dispensed Rx [DOT], that represents the Days of Therapy (DOT) dispensed to patients.

Days of therapy (DOT) is a measure defining the number of therapy days (according to Defined Daily Dosages, DDD) ensured by the number of packages of a given medication sold in a given period of time. It is assumed that Defined Daily Dosages (DDD) correspond to the WHO guidelines defining the recommended daily dosage for each therapeutic substance. DOT is calculated by converting dispensed prescriptions (Rx) into the corresponding number of treatment days, based on unit strength (dose), dosing regimen, and pack size.

This metric most accurately reflects real treatment exposure and is therefore the closest measure for analyses of adherence and consumption. The national-level IQVIA Pharmascope dataset describes all products reimbursed in community pharmacy in Poland. We filtered a data set from 01/2018 to 12/2024 (monthly) on drugs with the following codes ICD F32 and F32. 0, F32. 1, F32,2, F32. 3, F34, F32. 8, F32. 9, F33, F33. 1, F33. 2, F33. 3, F33. 4, F33. 8, F33. 9, F38, F39, F43,20 F43, 21 F40, F40. 0, F40. 1, F40. 2, F40. 8, F40. 9 F41, F41. 0, F41. 2, F41. 3, F41. 8, F41. 9, F93. 0, F93. 1, F93. 2 (see **Table 1**).

**Table 1:** List of ICD-10 diagnostic codes for mood and anxiety disorders.

ICD-10 code	Disease name
F32	Depressive episode
F32.0	Mild depressive episode
F32.1	Moderate depressive episode
F32.2	Severe depressive episode without psychotic symptoms
F32.3	Severe depressive episode with psychotic symptoms
F32.8	Other depressive episodes
F32.9	Depressive episode, unspecified
F33	Recurrent depressive disorder
F33.1	Recurrent depressive disorder, current episode moderate
F33.2	Recurrent depressive disorder, current episode severe without psychotic symptoms
F33.3	Recurrent depressive disorder, current episode severe with psychotic symptoms
F33.4	Recurrent depressive disorder, currently in remission
F33.8	Other recurrent depressive disorders
F33.9	Recurrent depressive disorder, unspecified
F34	Persistent mood (affective) disorders
F38	Other mood (affective) disorders
F39	Unspecified mood (affective) disorder
F40	Phobic anxiety disorders
F40.0	Agoraphobia
F40.1	Social phobias
F40.2	Specific (isolated) phobias
F40.8	Other phobic anxiety disorders
F40.9	Phobic anxiety disorder, unspecified
F41	Other anxiety disorders
F41.0	Panic disorder (episodic paroxysmal anxiety)
F41.2	Mixed anxiety and depressive disorder
F41.3	Other mixed anxiety disorders
F41.8	Other specified anxiety disorders
F41.9	Anxiety disorder, unspecified
F43.20	Adjustment disorder, unspecified
F43.21	Adjustment disorder with depressed mood
F93.0	Separation anxiety disorder of childhood
F93.1	Phobic anxiety disorder of childhood
F93.2	Social anxiety disorder of childhood

Drugs that were analyzed both in depression and anxiety were: sertraline, fluoxetine, fluvoxamine, citalopram, escitalopram, trazodone, venlafaxine; only in depression: tianeptine, mianserine. Also, in children and adolescents, it is not uncommon for anxiety disorders as well as depressive disorders with a strong anxiety component to use hydroxyzine, alprazolam, lorazepam, diazepam, chlorprothixene and quetiapine, so data for this drug was also analyzed (see **Table 2**).

**Table 2:** Analyzed drugs use.

Drug name	Notes
Fluoxetine	Selective Serotonin Reuptake Inhibitor
Fluvoxamine	Selective Serotonin Reuptake Inhibitor
Citalopram	Selective Serotonin Reuptake Inhibitor
Escitalopram	Selective Serotonin Reuptake Inhibitor
Sertaline	Selective Serotonin Reuptake Inhibitor
Trazodone	Often for sleep, sedation
Venlafaxine	Serotonin-Norepinephrine Reuptake Inhibitor
Tianeptine	Atypical antidepressant
Mianserine	Tetracyclic antidepressant
Hydroxyzine	Antihistamine with anxiolytic properties
Alprazolam	Benzodiazepine
Lorazepam	Benzodiazepine
Diazepam	Benzodiazepine
Chlorprothixene	Typical antipsychotic, sedating
Quetiapine	Atypical antipsychotic, off-label anxiolytic

We have further examined the combined annual number of sick-leave days for 2020–2024 pertaining to the ICD-10 codes F32. 0–F32. 3, F32. 8–F32. 9, F33. 0–F33. 9, F34, F38–F39 (recurrent depressive episode and depressive episodes) (dysthymic and other mood disorder), F40. 0–F40. 2, F40. 8–F40. 9, F41. 0, F41. 2–F41. 3, F41. 8–F41. 9 (anxiety disorders), F43. 20–F43. 21 (Disorders of stress), and F93. 0–F93. 2 (childhood emotional disturbances) (see **Table 1**). Annual data were obtained directly from the Social Insurance Institution of Poland (pol. Zakład Ubezpieczeñ Społecznych, ZUS). The Social Insurance Institution (ZUS) is a central governmental agency in Poland responsible for the assessment of entitlement to social benefits, for the collection of social security contributions (including retirement, disability, sickness and healthcare premiums), and for the payment of such benefits, among other responsibilities, it compiles the total number of days within absence cases due to the respective ICD-10 codes. It is important to note that the analyzed ZUS data represent caregiver sick leave days issued to parents or legal guardians caring for a child with a mental health diagnosis, not school absences of children or work absences of adolescents. These administrative records reflect the burden on caregivers and the healthcare system rather than direct absenteeism among children.

Lastly, we considered the dataset consisting of annual counts of children aged less than 18 years of age who received at least one intervention in psychiatric care, addiction treatment or dedicated intervention program during the years 2018—2024 as defined by ICD-10 F32. 0–F32. 3, F32. 8–F32. 9, F33. 0–F33. 9, F34, F38–F39, F40. 0–F40. 2, F40. 8–F40. 9, F41. 0, F41. 2–F41. 3, F41. 8–F41. 9, F43. 20–F43. 21, and F93. 0F93. 2. Annual numbers of patients were derived from country-level health records by seeking out all unique persons aged < 18 years who had at least 1 service of the type and ICD-10 code. Rate of change, year-to-year was determined to evaluate changes in service use.

### 2.1 Handling of the 2019 reporting discontinuity.

In August 2019, Poland introduced a three-tier model of child and adolescent mental health, creating Level-I community-based psychological and psychotherapeutic centers as separate benefit categories (18). Administrative guidance and NFZ communications indicate distinct contracting and reporting streams for Level-I services. Given this system change, we prespecified 2019 as a potential structural break in the series of “treated under-18 psychiatric patients”. In primary analyses we included 2019 with a binary indicator (break_2019 = 1) and tested joinpoint/segmented regression allowing different intercepts/slopes pre- and post-2019. As a robustness check, we repeated all longitudinal models excluding 2019.

### 2.2 Statistical analysis

The collected data concerning both the analyzed medications and sick leaves were statistically analyzed. A total number of dispensed Rx (measured in DOT) for each medication in months between January 2018 and December 2024 is illustrated together with a trend line in the figures in the results section. Data on sick leaves and medical consultations were illustrated in a similar way in figures. Linear regression was calculated to assess the relationship between prescribed medications and time period (including and excluding the lockdown period). Additionally, a modified Gompertz model was used to mathematically describe the drug’s unit (DOT) accumulation (for all analyzed medications). This is described in detail in the results section.

## 3 Results

### 3.1 Medication/drug use

**Figure 2:**
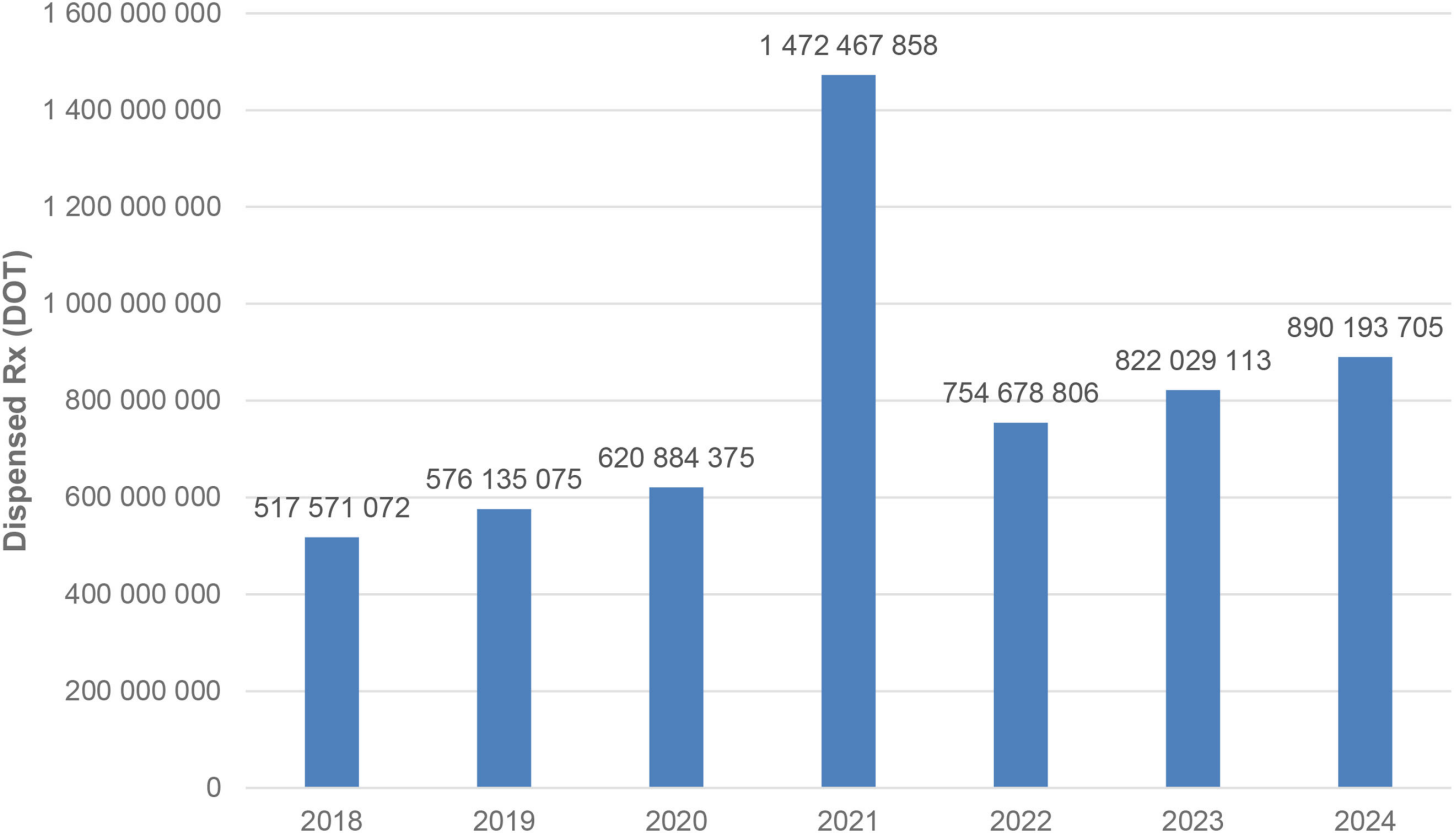
All analyzed drugs (medications) used in children and adolescents in Poland.

**Figure 3:**
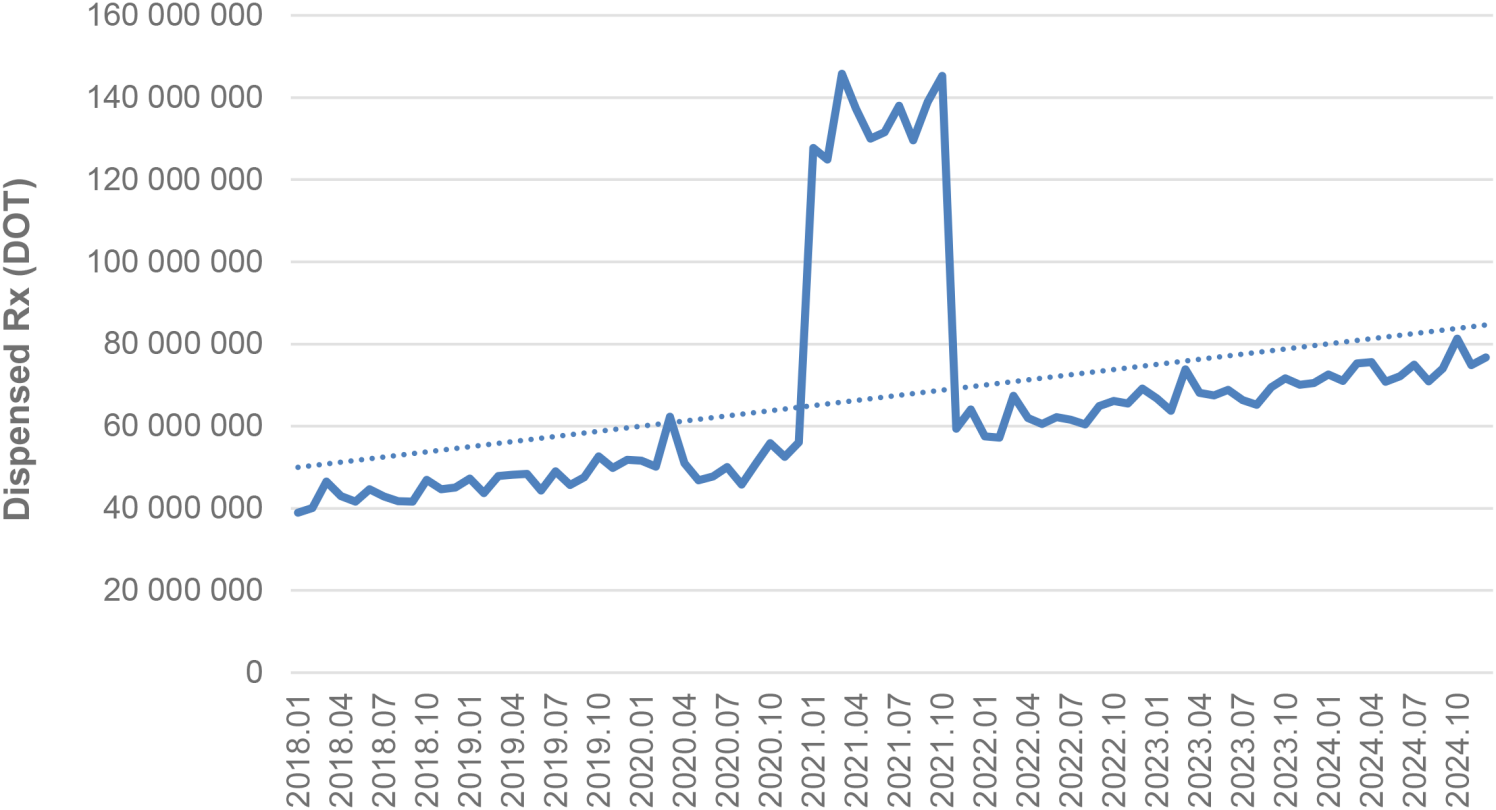
All analyzed drugs (medications) use in children and adolescents in Poland.

**Figure 4:**
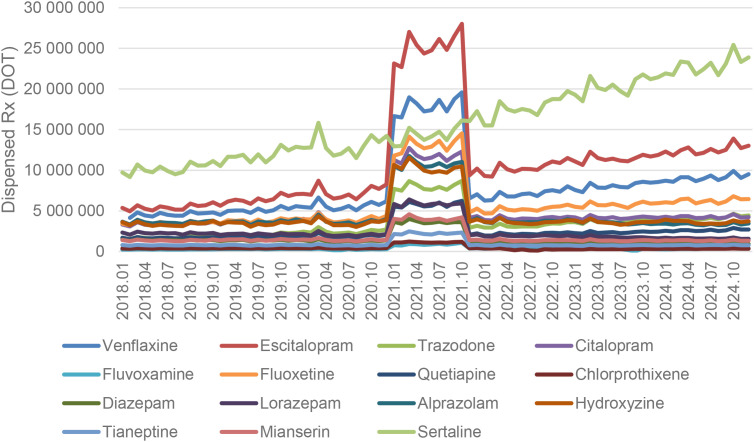
All analyzed drug (medications) use in children and adolescents in Poland.

Between January 2018 and December 2024, a total of psychotropic medication dispensed DOT in Poland rose steadily from 517.6 million in 2018 to 620.9 million in 2020, risen sharply to 1 472.5 million in 2021 (the height of COVID-19 restrictions), then declined to 754.7 million in 2022 and continued to rise, reaching about 890.2 million in 2024 (see **Figures 2**–**4**).

#### 3.1.1 Selective serotonin reuptake inhibitors.

**Figure 5:**
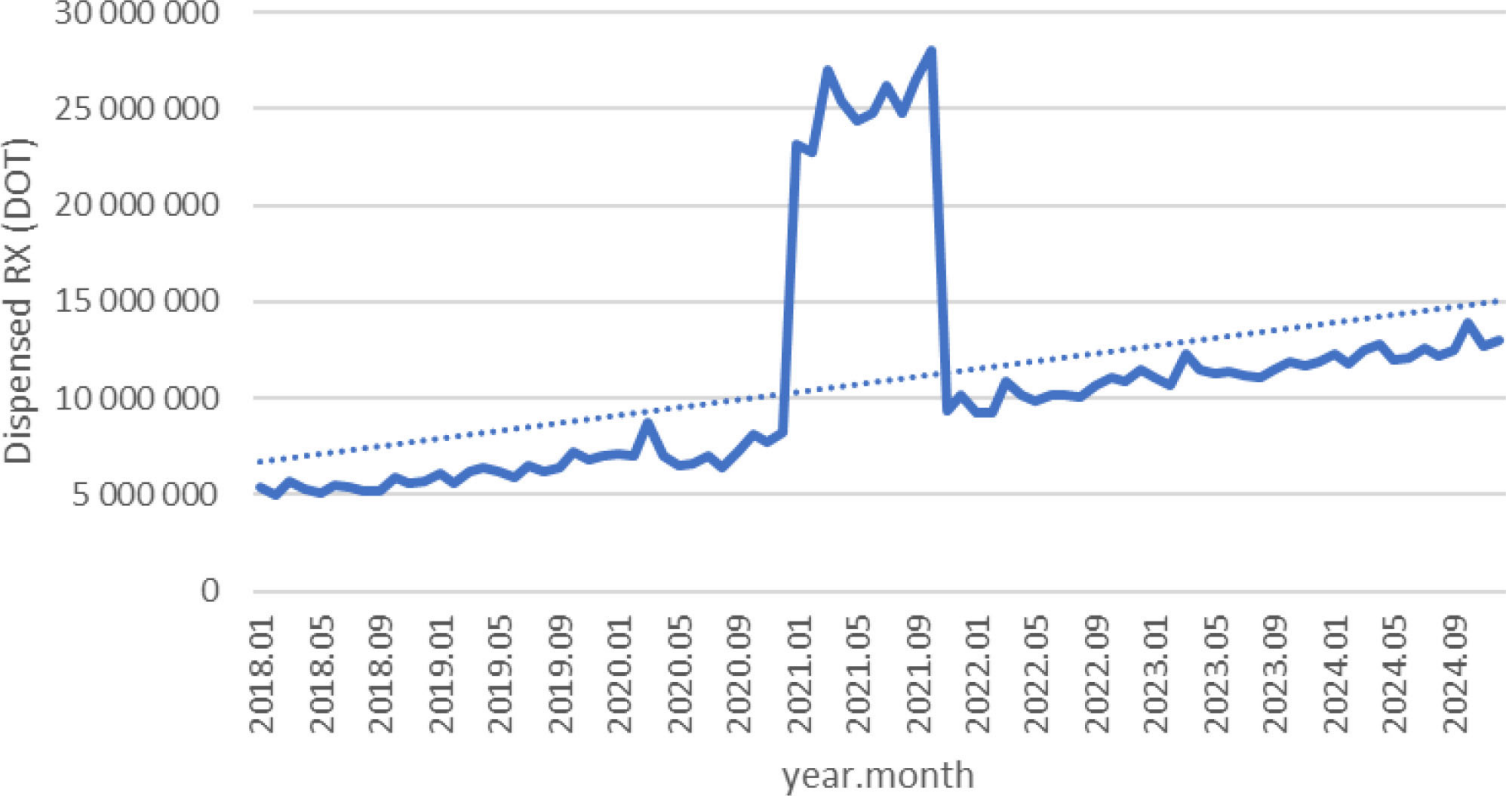
Escitalopram use in children and adolescents in Poland.

Escitalopram’ dispensed therapy days (DOT) increased from around 5.5 million in 2018 to 7.5 million in 2020, peaked at nearly 27 million in 2021, then returned to approximately 10 million in 2022 and continued to rise, reaching about 13 million in 2024 (see **Figure 5**).

**Figure 6:**
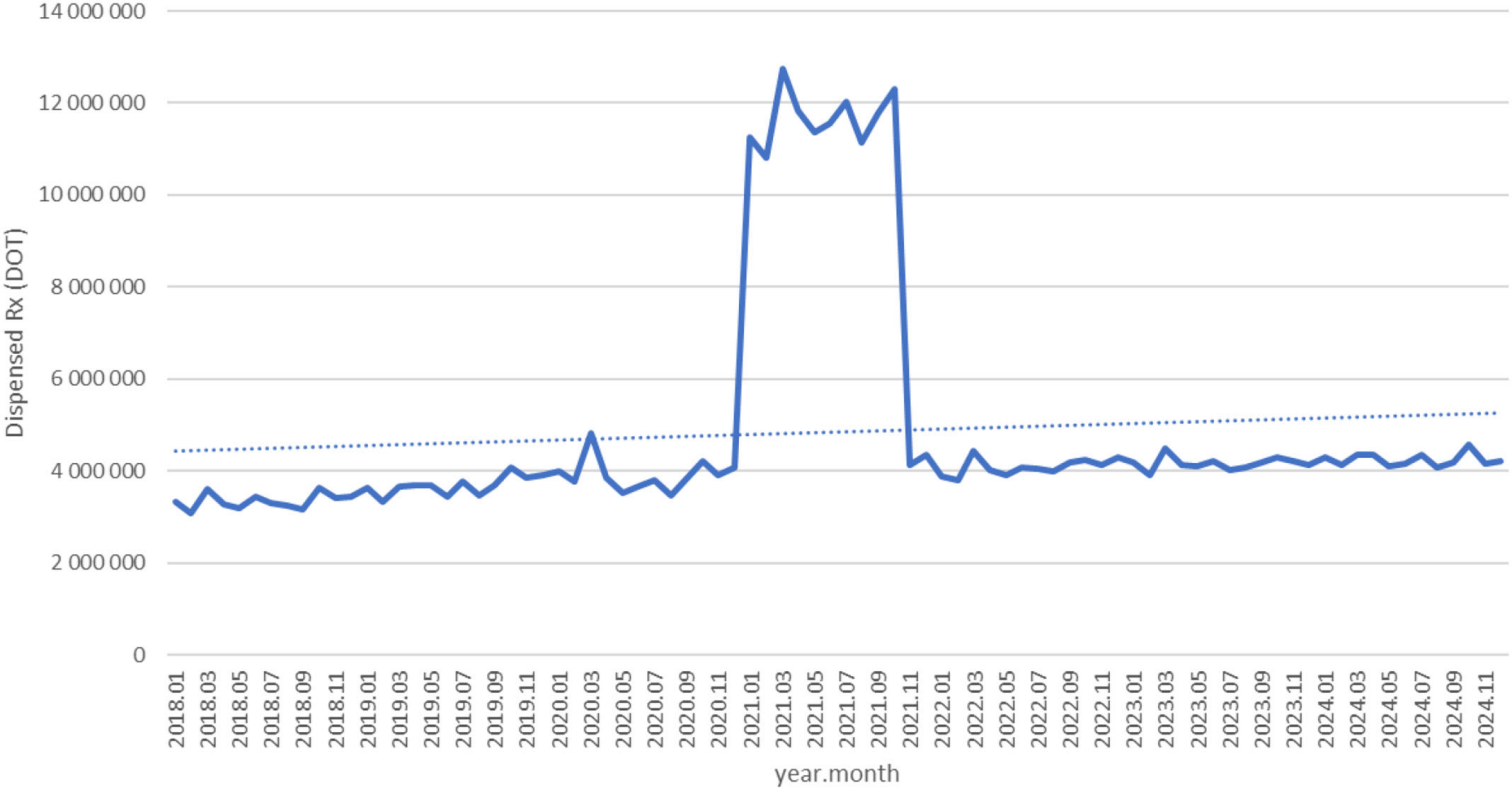
Citalopram use in children and adolescents in Poland.

Dispensed therapy days (DOT) for citalopram increased from about 3.2 million in 2018 to 3.8 million in 2020, spiked to nearly 12.5 million in 2021, then declined to around 4 million in 2022 and remained stable with a slight upward drift through 2024 (see **Figure 6**).

**Figure 7:**
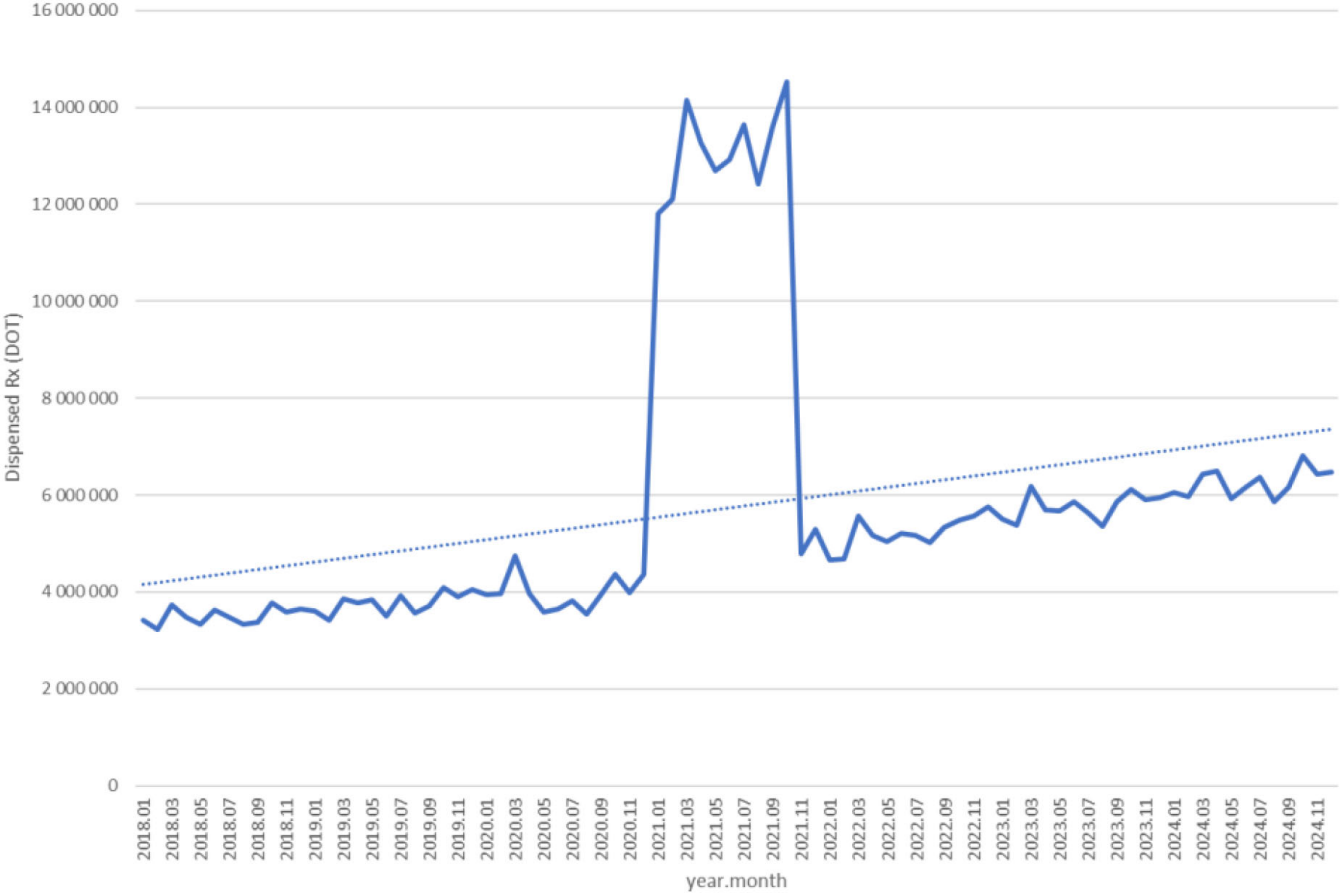
Fluoxetine use in children and adolescents in Poland.

Dispensed therapy days for fluoxetine increased from roughly 3.3 million in 2018 to about 4.0 million in 2020, surged to nearly 15 million in 2021, then fell back to around 4.5 million in 2022 and continued a steady rise, reaching approximately 6.5 million in 2024 (see **Figure 7**).

**Figure 8:**
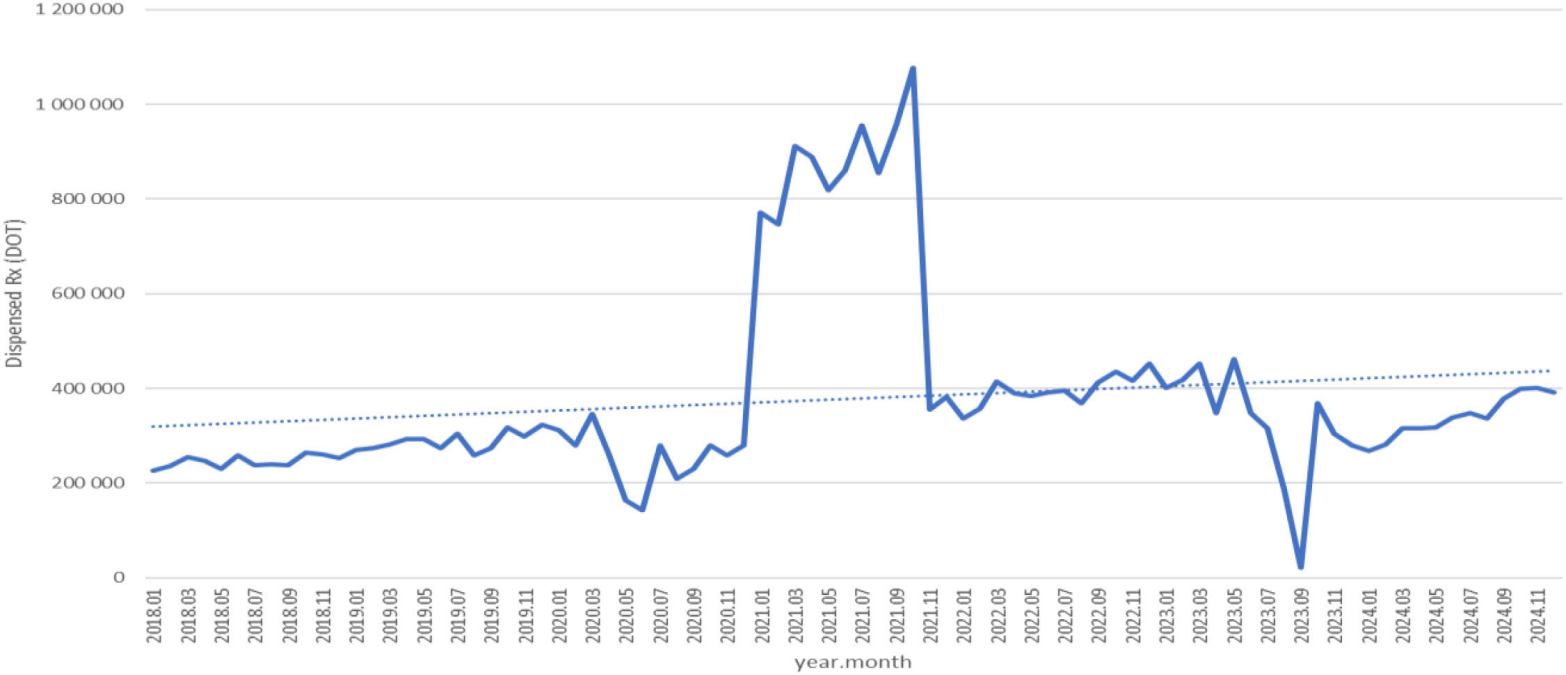
Fluvoxamine use in children and adolescents in Poland.

Dispensed therapy days for fluvoxamine increased from about 230,000 in 2018 to roughly 320,000 in 2020, rose sharply to nearly 1.1 million in 2021, then fell back to around 360,000 in 2022 and continued to fluctuate modestly, reaching approximately 400,000 in 2024 (see **Figure 8**).

**Figure 9:**
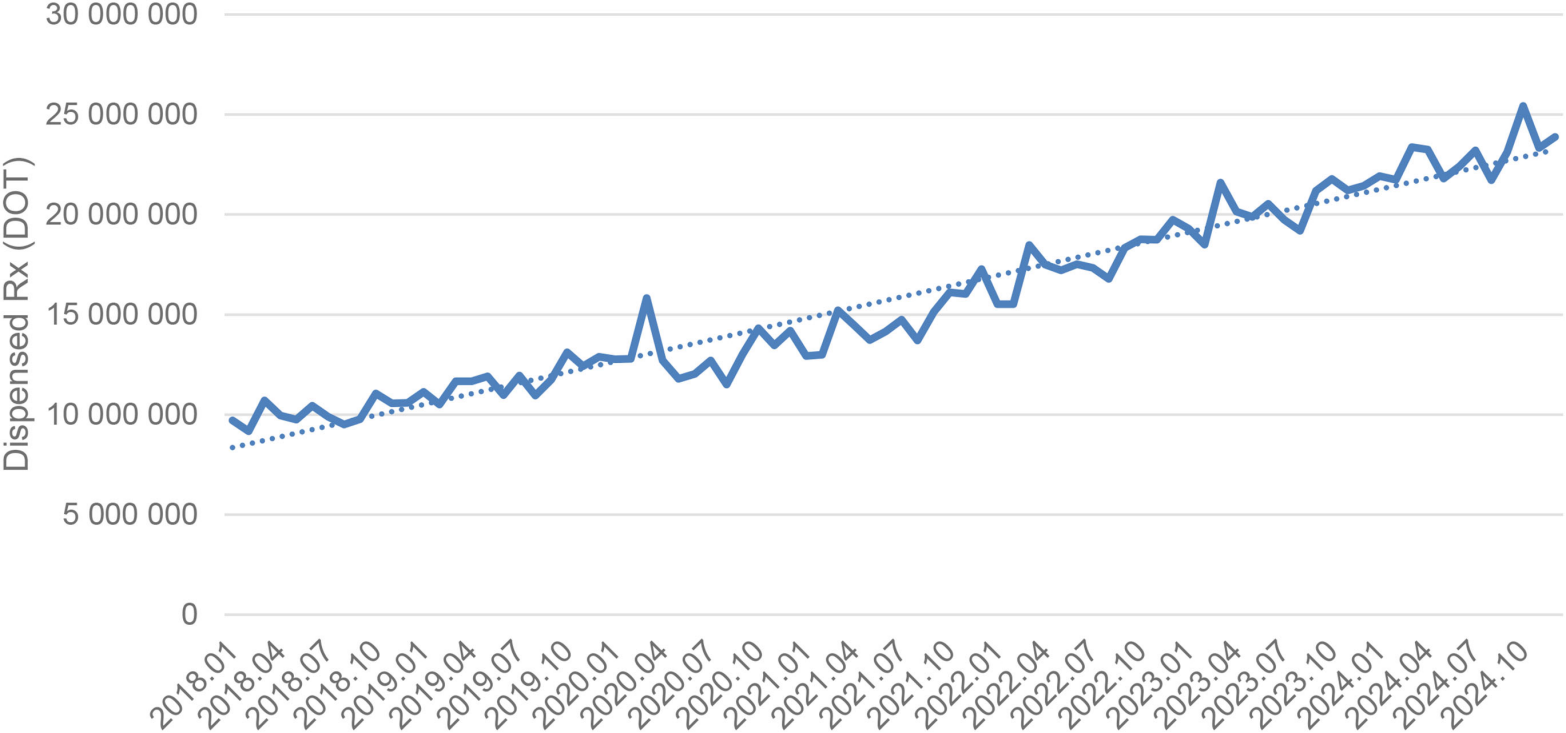
Sertaline use in children and adolescents in Poland.

Dispensed therapy days for sertaline increased steadily from about 9.5 million in 2018 to roughly 14 million in 2020, rose further to around 18 million in 2022, and continued climbing to approximately 23–25 million by 2024, following a strong and consistent upward trend (see **Figure 9**).

#### 3.1.2 Serotonin and norepinephrine reuptake inhibitors

**Figure 10:**
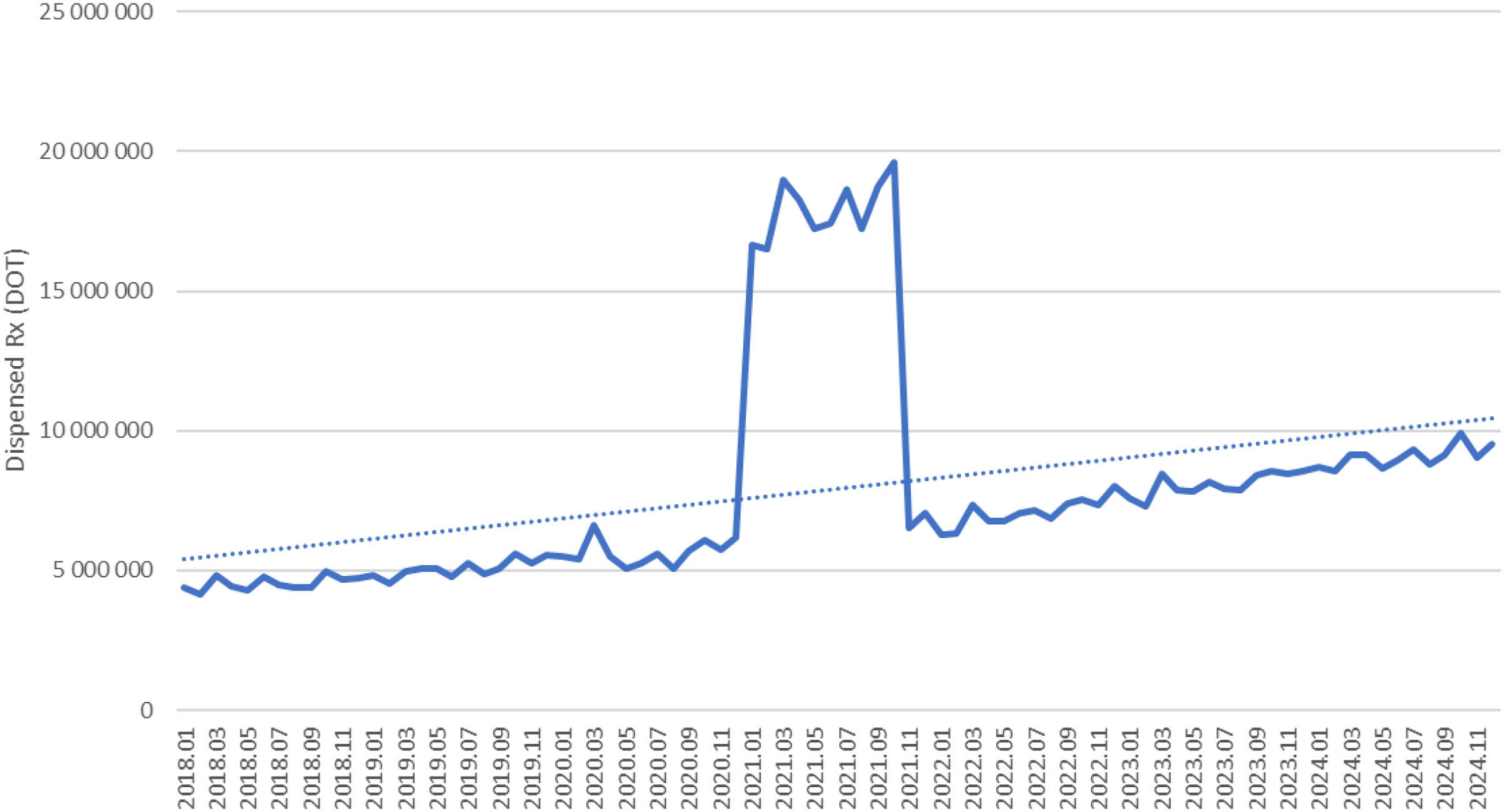
Venlafaxine use in children and adolescents in Poland.

Dispensed therapy days for venlafaxine increased from about 4.3 million in 2018 to approximately 5.5 million in 2020, surged to nearly 19 million in 2021, then declined to around 6.5 million in 2022 and continued to rise steadily, reaching roughly 9.5 million in 2024 (see **Figure 10**).

#### 3.1.3 Other antidepressants

**Figure 11:**
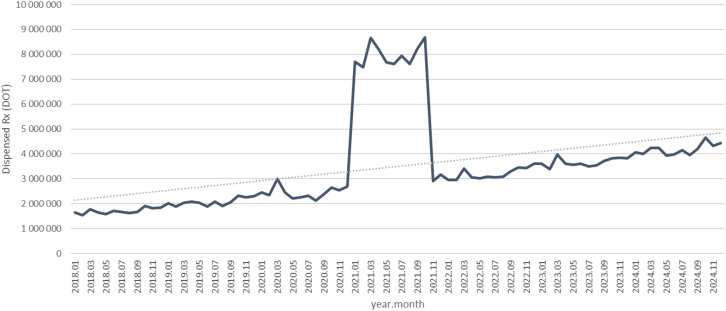
Trazodone use in children and adolescents in Poland.

As you can see on **Figure 10**. trazodone use in dispensed therapy days (DOT) increased from about 1.6 million in 2018 to roughly 2.3 million in 2020, rose sharply to nearly 9 million in 2021, then declined to around 3 million in 2022 and continued a steady upward trend, reaching approximately 4.5 million in 2024 (see **Figure 11**).

**Figure 12:**
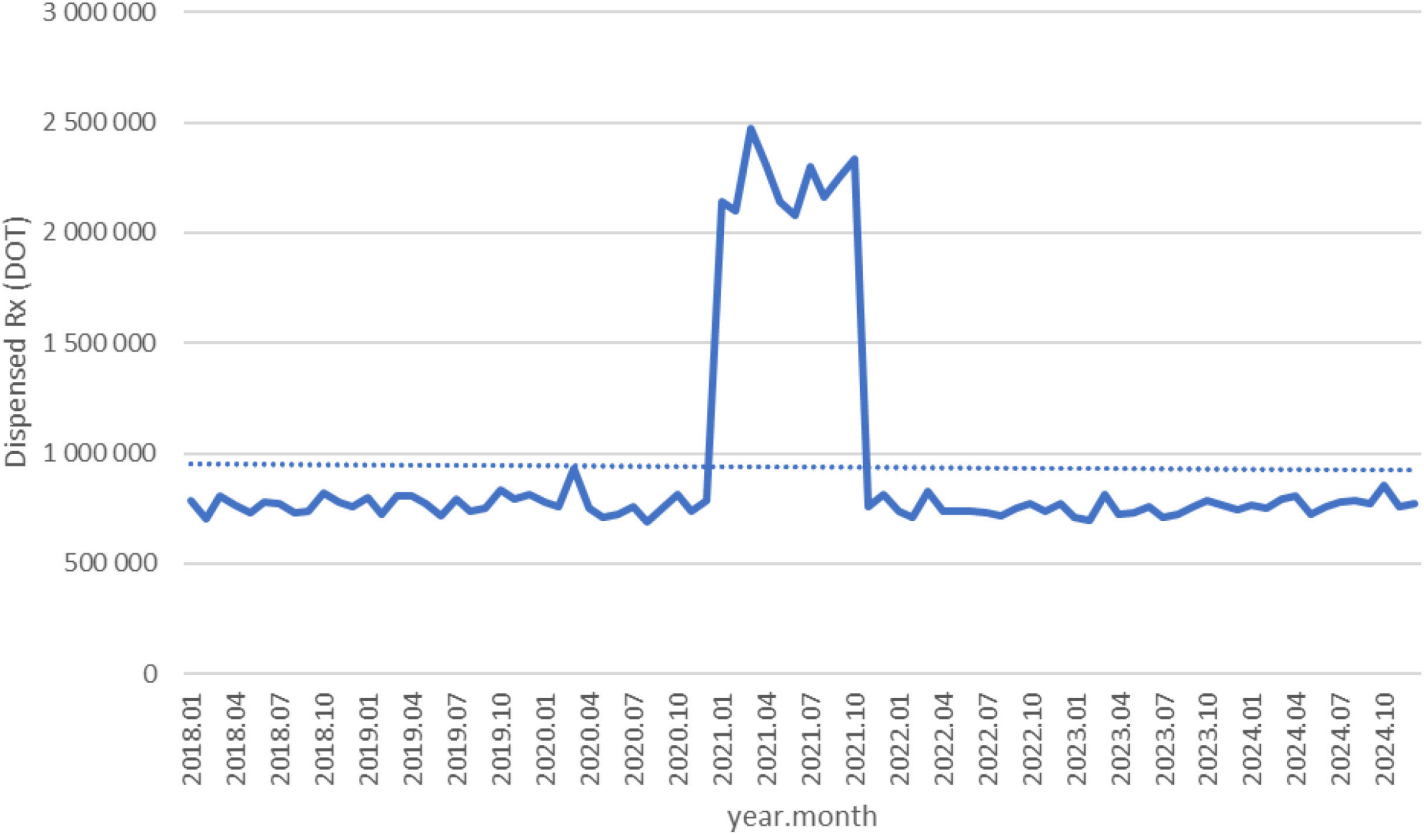
Tianeptine use in children and adolescents in Poland.

**Figure 13:**
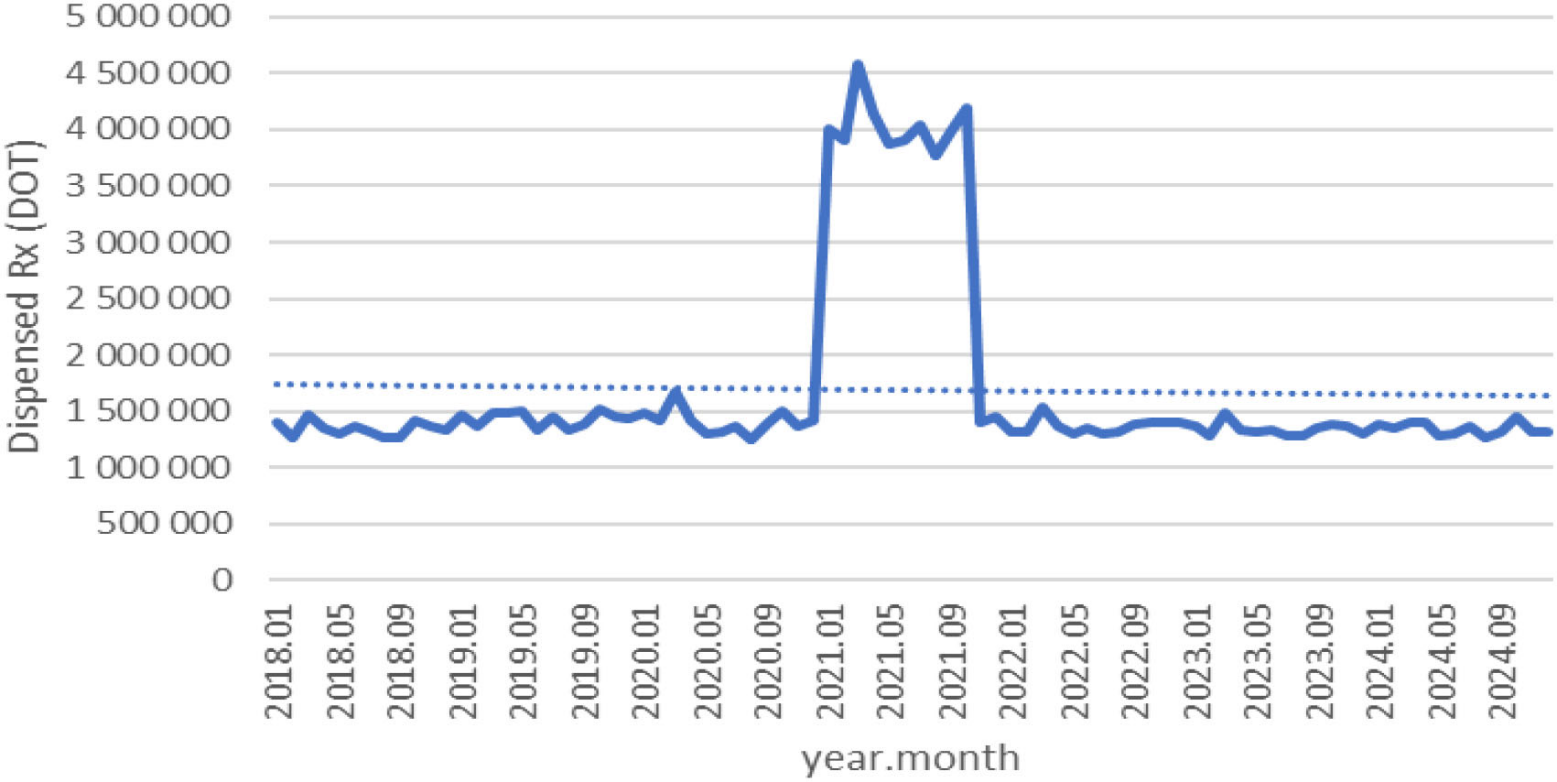
Mianserin use in children and adolescents in Poland.

Tianeptine and mianserin showed modest fluctuations and did not exhibit sustained post-pandemic increases (see **Figures 12**. and **13**). DOT for Tianeptine increased from about 750,000 in 2018 to roughly 850,000 in 2020, spiked to nearly 2.4 million in 2021, then returned to around 800,000 in 2022 and remained stable through 2024 (see **Figure 12**). For Mianserin DOT increased from about 1.3 million in 2018 to roughly 1.5 million in 2020, spiked to nearly 4.2 million in 2021, then fell back to around 1.4 million in 2022 and remained stable through 2024 (see **Figure 13**).

#### 3.1.4 Anxiolytics (benzodiazepines and related agents)

**Figure 14:**
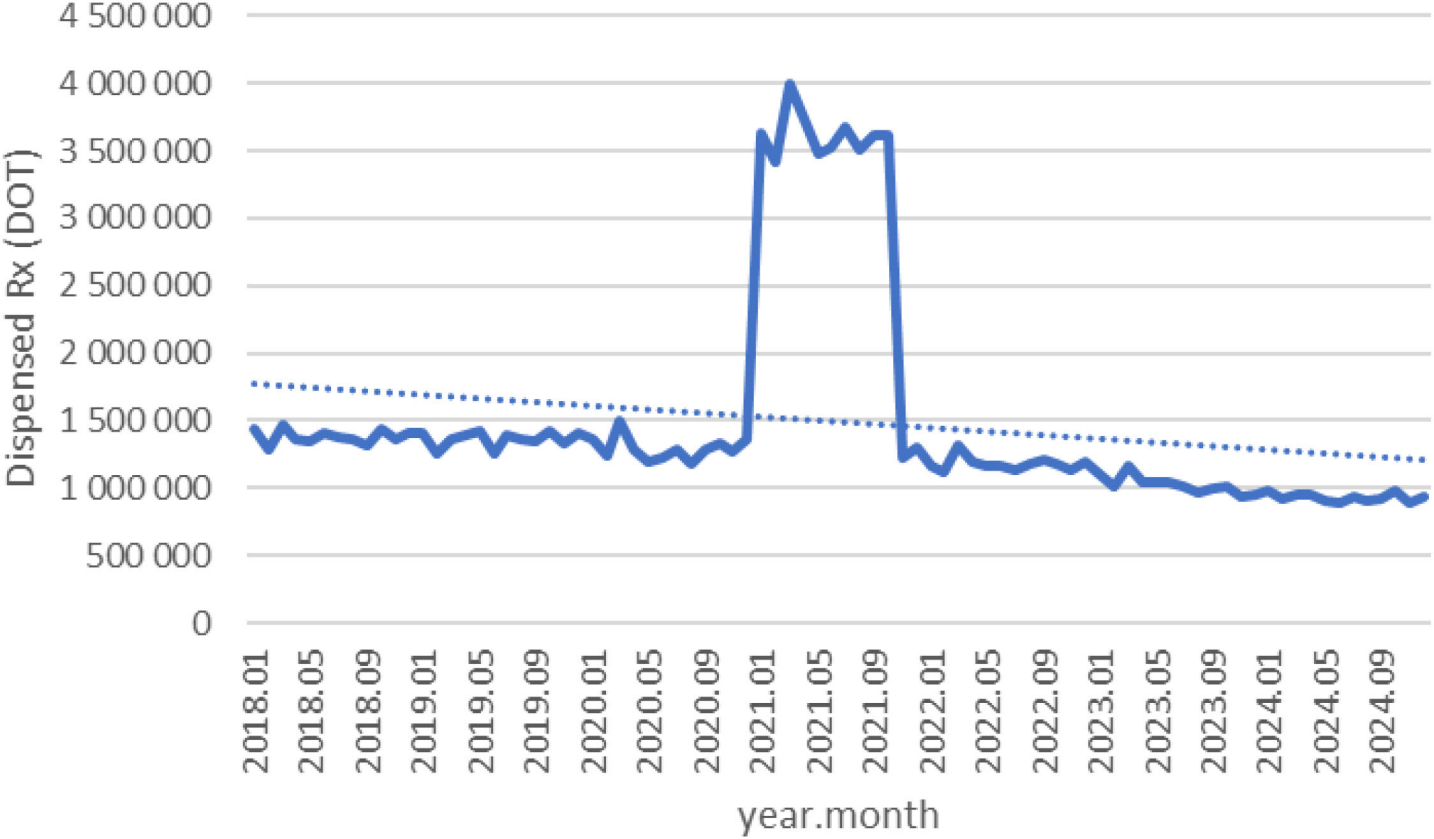
Diazepam use in children and adolescents in Poland.

Diazepam declined gradually from about 1.45 million in 2018 to roughly 1.3 million in 2020, spiked to nearly 4.0 million in 2021, then fell back to around 1.2 million in 2022 and continued a slow downward trend to approximately 1.1 million in 2024 (see **Figure 14**).

**Figure 15:**
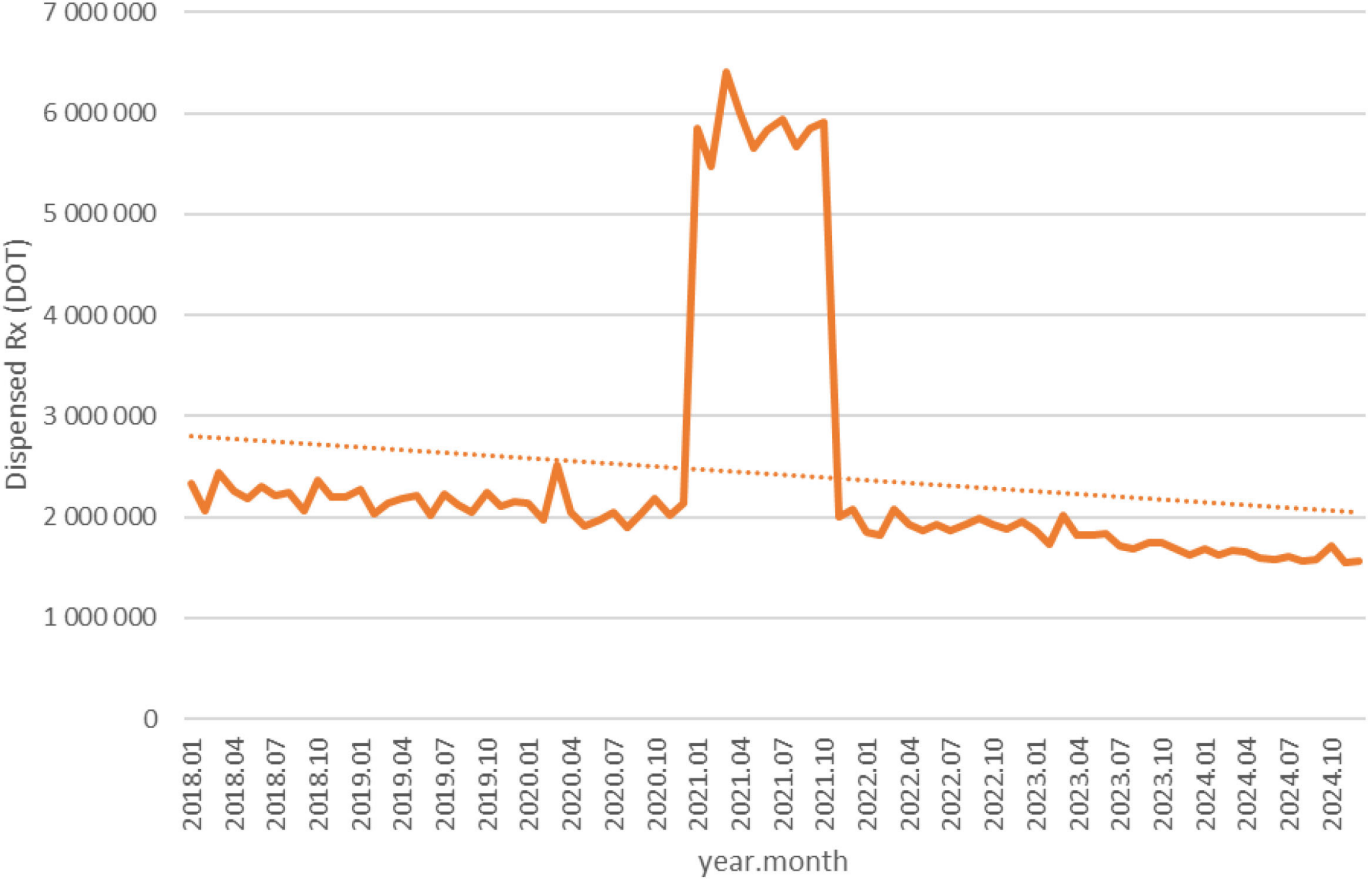
Lorazepam use in children and adolescents in Poland.

Lorazepam dispensing declined from about 2.2 million in 2018 to roughly 2.0 million in 2020, spiked to nearly 6.6 million in 2021, then fell back to around 1.9 million in 2022 and continued a gradual downward trend to approximately 1.6 million in 2024 (see **Figure 15**).

**Figure 16:**
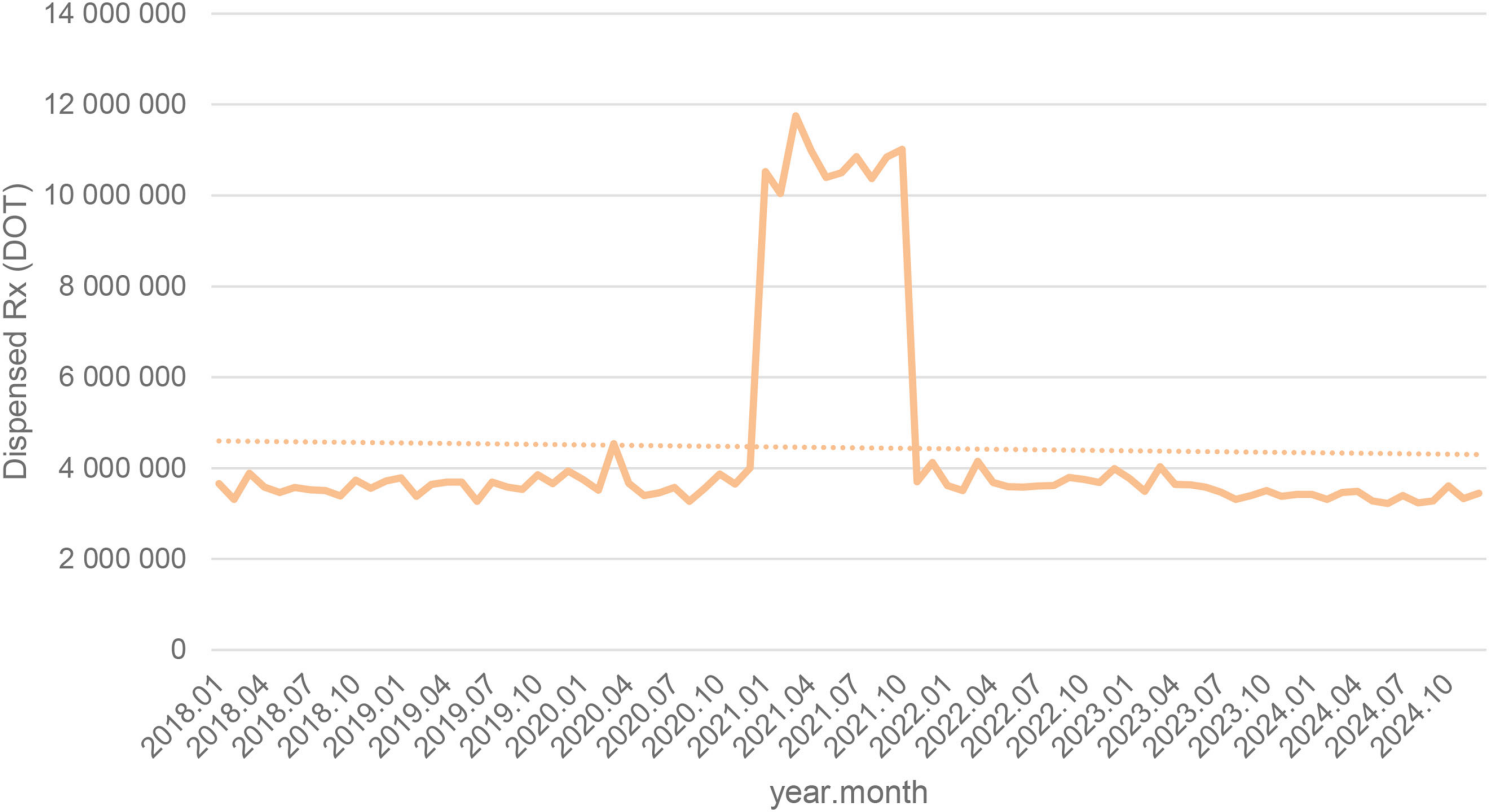
Alprazolam use in children and adolescents in Poland.

Before the pandemic, alprazolam dispensing remained stable at around 3.5–4.0 million between 2018 and 2020, surged to nearly 12 million in 2021, then declined to approximately 3.7 million in 2022 and continued a gradual downward trend to about 3.3 million in 2024 (see **Figure 16**).

**Figure 17:**
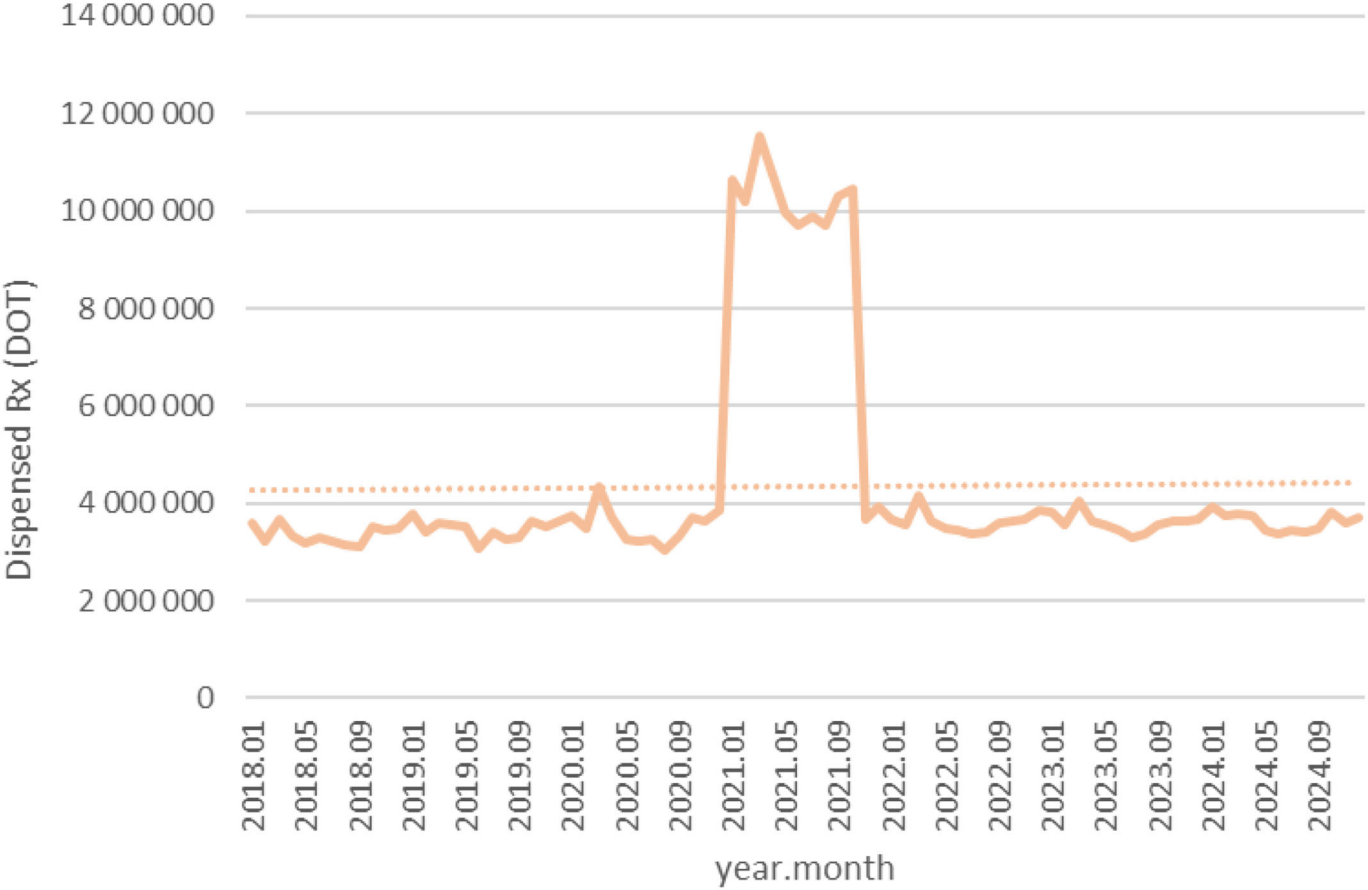
Hydroxyzine use in children and adolescents in Poland.

Although hydroxyzine isn’t a benzodiazepine, its dispensing followed a similar trajectory rising from 3.4 million DOT in 2018 to roughly 3.7 million in 2020, surged to nearly 11.5 million in 2021, then declined to around 3.8 million in 2022 and remained broadly stable through 2024 (see **Figure 17**).

#### 3.1.5 Antipsychotics

**Figure 18:**
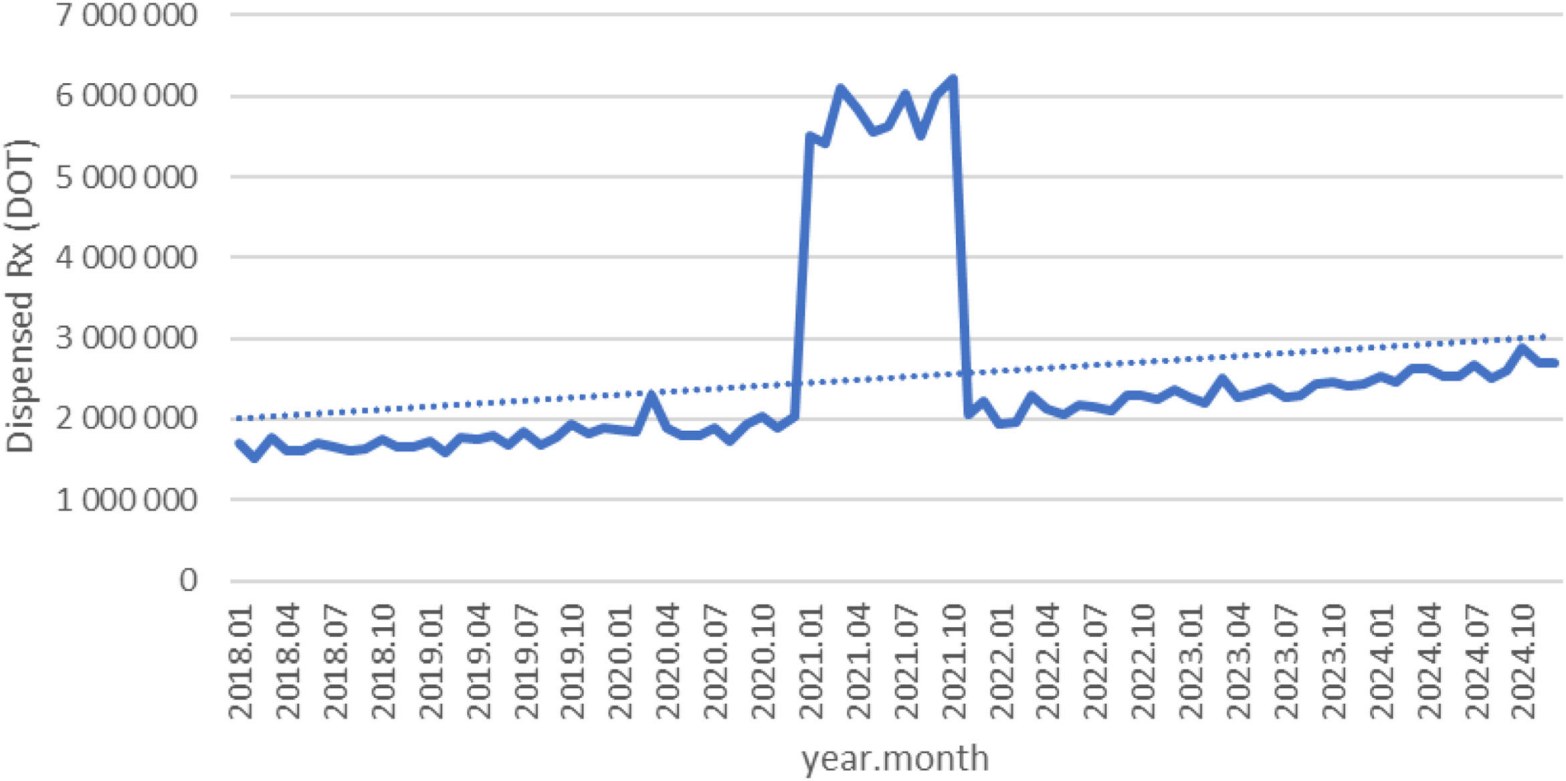
Quetiapine use in children and adolescents in Poland.

Quetiapine DOT as seen on **Figure 17**. increased from about 1.7 million in 2018 to roughly 2.0 million in 2020, rose sharply to nearly 6.2 million in 2021, then declined to around 2.1 million in 2022 and continued a steady upward trend, reaching approximately 2.7 million in 2024 (see **Figure 18**).

**Figure 19:**
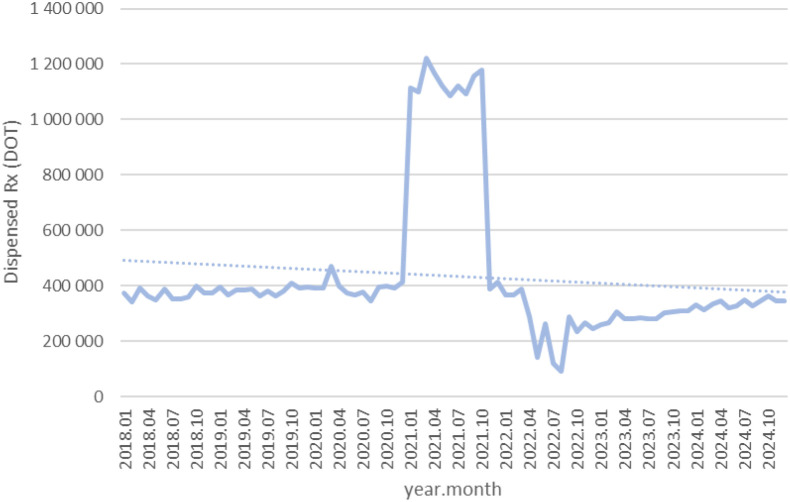
Chlorprothixene use in children and adolescents in Poland.

Chlorprothixene dispensing remained stable at around 360,000–390,000 DOT between 2018 and 2020, surged to nearly 1.2 million in 2021, then fell sharply to about 250,000 in 2022 and gradually recovered to roughly 350,000 by 2024 (see **Figure 19**).

Overall, almost all agents (except sertraline) exhibited a pronounced spike in 2021 coinciding with the COVID-19 pandemic, followed by declines in 2022–2024. However, several antidepressants (notably escitalopram, fluoxetine. venlafaxine, trazodone) and quetiapine remained substantially above their 2018–2019 dispensing levels by the end of 2024, whereas most anxiolytics reverted to or fell below pre-pandemic use. The exceptional case seems to be sertraline, which did not show a clear peak, but rather consistent dynamic growth over the years analyzed.

### 3.2 Sick leaves and patients treated

**Figure 20:**
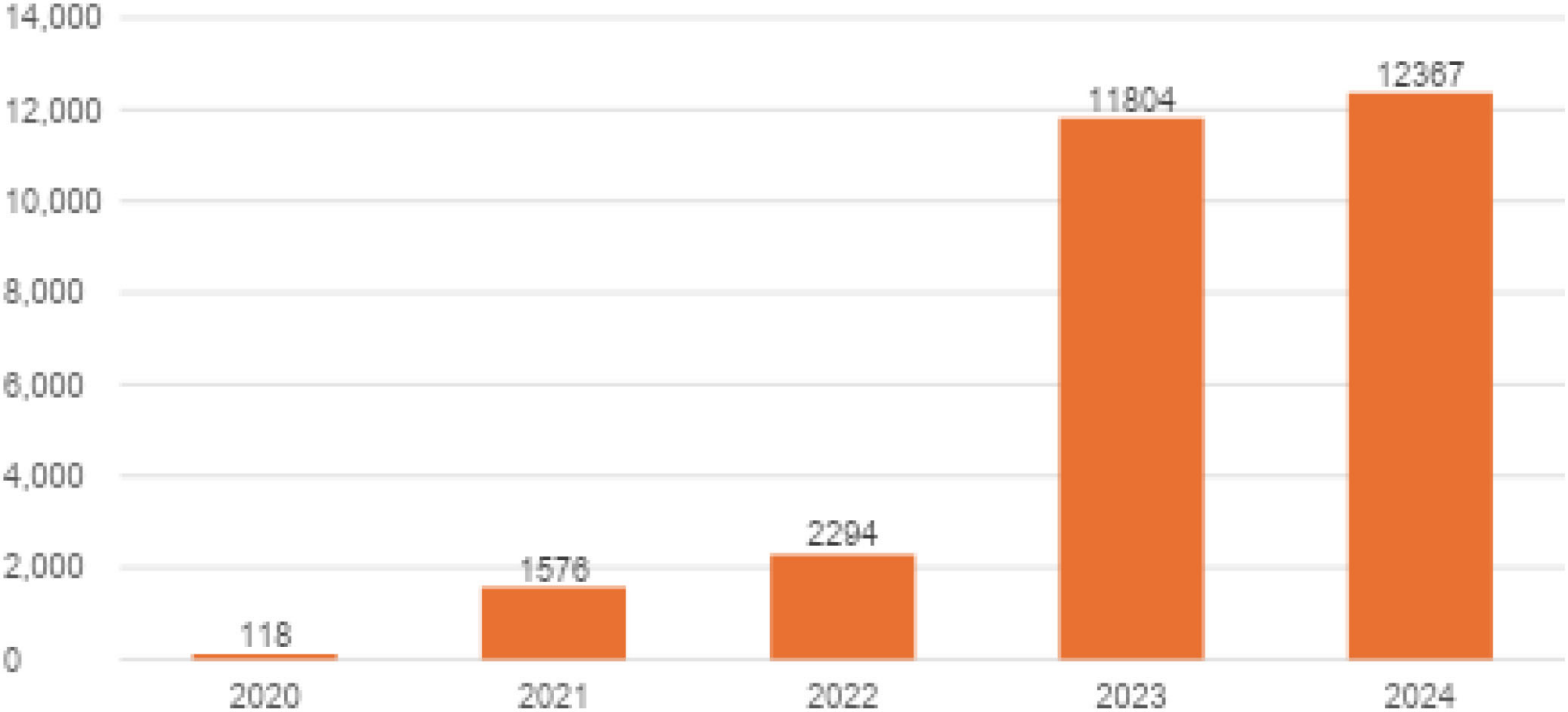
Yearly totals for sick-leave days due to ICD-10 codes F32.0–F32.3, F32.8–F32.9, F33.0–F33.9, F34, F38–F39, F40.0–F40.2, F40.8–F40.9, F41.0, F41.2–F41.3, F41.8–F41.9, F43.20–F43.21, and F93.0–F93.2 in Poland.

Regarding the sick-leave due to ICD-10 codes F32.0–F32.3, F32.8–F32.9, F33.0–F33.9 (depressive episodes and recurrent depression), F34, F38–F39 (dysthymia and other mood disorders), F40.0–F40.2, F40.8–F40.9, F41.0, F41.2–F41.3, F41.8–F41.9 (anxiety disorders), F43.20–F43.21 (stress-related disorders), and F93.0–F93.2 (childhood emotional disorders), the yearly totals of sick-leave days were 118 for 2020, 1–576 for 2021, 2–294 for 2022, 11–804 for 2023, and 12–367 for 2024 (see **Figure 20**).

In 2020 and 2021, the number of sick-leave days soared. Afterward, it continued to increase at a slower rate until 2022 (reaching 2294 days) before another steep jump in 2023 (11–804 days). The 2024 value presents a mild additional increase, but then it levels off at 12367 days.

**Figure 21:**
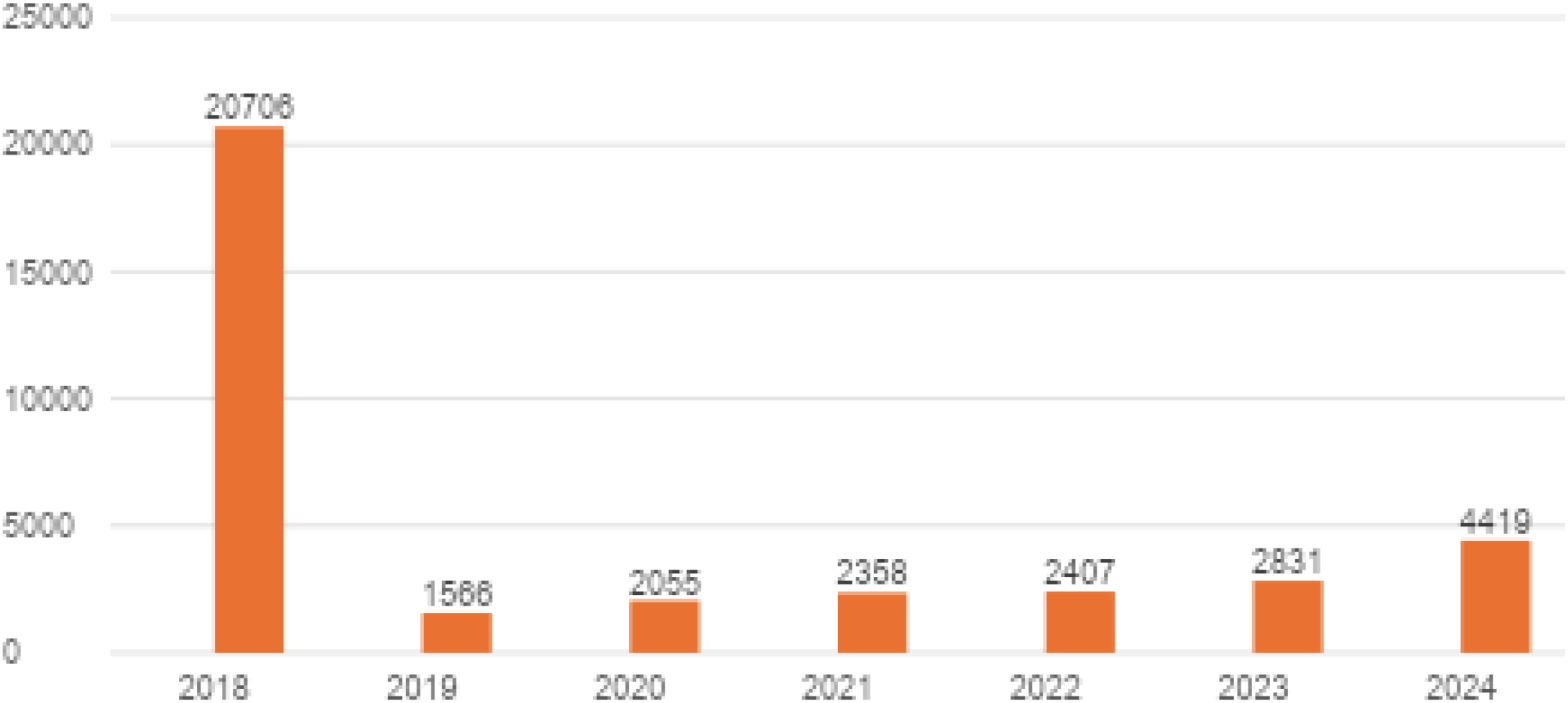
Annual number of patients under 18 receiving psychiatric or addiction treatment for ICD codes F32.0–F32.3, F32.8–F32.9, F33.0–F33.9, F34, F38–F39, F40.0–F40.2, F40.8–F40.9, F41.0, F41.2–F41.3, F41.8–F41.9, F43.20–F43.21, and F93.0–F93.2 in Poland.

In relation to patients treated, 20–706 under 18-year-olds were treated in 2018, but this figure fell steeply to 1–566 in 2019. Because in August 2019 the Ministry of Health launched the new three-tier architecture of child and adolescent mental health care (18), with Level-I community psychological and psychotherapeutic centers being reported under distinct benefit categories and contracting pathways (separate from classical child psychiatry units), this year constitutes a structural reporting break. In our primary longitudinal models we therefore coded 2019 as a binary break indicator and, as recommended, we additionally performed sensitivity analyses excluding 2019; conclusions for 2020–2024 trends remained directionally robust. Counts then increased slowly to 2055 in 2020, 2358 in 2021 and 2407 in 2022, and then to 2831 in 2023 and 4419 in 2024. This is a rise from 2022–24 and is maintained after passing through the post-2018 trough (**Figure 21**).

### 3.3 Linear regression

In the statistical analysis of drug use among children and adolescents in Poland, data from the early lockdown period (primarily the first half of 2020) were excluded from the main time-trend models. This decision was made to preserve the interpretive clarity and structural continuity of the long-term trajectories rather than to amplify any effect. The lockdown months represented an exceptional, system-wide disruption, characterized by abrupt restrictions on in-person consultations, irregular prescription renewals, temporary shortages, and substantial instability in healthcare delivery. Including such an anomalous interval introduced non-replicable distortions that severely reduced model fit for instance, for venlafaxine, the coefficient of determination dropped to R² = 0.1351, indicating that only about 14% of the variance could be explained by a linear trend, whereas after exclusion it improved to R² = 0.9545.

By omitting this highly atypical period, the analysis better reflects stable prescribing dynamics in pre- and post-pandemic conditions, thereby enhancing the transparency and validity of the time-series interpretation. Nevertheless, supplementary sensitivity checks including the lockdown months confirmed that the general direction of post-pandemic trends remained consistent, though with attenuated slopes. The slope coefficient for venlafaxine (a =62766) thus represents the average monthly increase under stabilized prescribing conditions.

**Table 3** summarizes the results of linear regression analyses for 15 psychotropic drugs commonly prescribed to children and adolescents in Poland between 2018 and 2024. For each compound, the coefficient of determination (R²) is presented both with and without inclusion of COVID-19 lockdown-period data, as well as the slope coefficient (rate of monthly change in drug usage) calculated using post-lockdown-adjusted data.

**Table 3:** Effect of COVID-19 lockdown on trend line fit and monthly usage dynamics of selected psychotropic drugs in the pediatric population in Poland (2018–2024).

Drug name	R² (with lockdown data)	R² (without lockdown data)	Coefficient for the independent variable (slope coefficient) – without lockdown data
Fluoxetine	0.1038	0.9305	40 010
Fluvoxamine	0.0285	0.2426	1 484.3
Citalopram	0.0089	0.6343	11 428
Escitalopram	0.1681	0.967	102 640
Trazodone	0.1823	0.9637	33 653
Venlafaxine	0.1351	0.9545	62 766
Tianeptine	0.0004	0.0053	-113
Mianserine	0.0012	0.0494	-661.76
Hydroxyzine	0.00004	0.1152	3 236.9
Alprazolam	0.0014	0.0577	-2 183.3
Lorazepam	0.0288	0.8061	-8 202.6
Diazepam	0.0421	0.8496	-6 307.4
Chlorprothixene	0.0161	0.2375	-1 233.6
Quetiapine	0.0555	0.9404	2 983.5
Sertaline*	0.9448	0.9565	179 227
All drugs	0.0483	0.9071	253 024

*Sertraline -lack of changes because of the lockdown

For most drugs analyzed, R² values were very low when lockdown data were included, indicating poor linear trend fit (e.g., fluvoxamine R² =0.0285, hydroxyzine R² = 0.00004). This suggests significant irregularity or volatility in prescribing during the early phase of the pandemic. After removing lockdown-period data, R² values markedly increased for several antidepressants and antipsychotics, indicating restoration of a consistent upward or downward prescribing trend (e.g., venlafaxine: from 0.1351 to 0.9545, quetiapine: from 0.0555 to 0.9404).

The highest positive slope coefficients reflecting consistent increases in usage—were observed for: escitalopram (+102640 units/month), venlafaxine (62–766 units/month) and sertraline (179227 units/month). These results are likely to reflect growing clinical adoption of these medications in pediatric psychiatry, possibly in response to rising rates of anxiety and depression post-2020.

Negative slope coefficients were observed for tianeptine, mianserine, chlorprothixene, alprazolam, lorazepam, and diazepam, suggesting either declining clinical relevance, tightening of prescribing practices, or safety concerns regarding use in younger populations. For example, diazepam use decreased by approximately 6–307 units/month, and lorazepam by over 8202 units/month.

Certain drugs, such as hydroxyzine and chlorprothixene, demonstrated persistently low R² values, even after removing lockdown data, implying highly variable, non-linear, or context-specific use likely linked to short-term symptomatic relief rather than chronic treatment plans. The significant increase in R² after exclusion of lockdown-period data highlights the necessity of adjusting time-series models for pandemic-related outliers. Including such anomalous periods may obscure meaningful trends and reduce the interpretability of linear models. This is particularly relevant in health technology assessments involving real-world evidence (RWE) and longitudinal pharmacoepidemiologic analyses.

### 3.4 Mathematical modeling

A modified Gompertz model was used to mathematically describe the drug’s unit (DOT) accumulation. This model was used to mathematically describe cancer cell growth and to mathematically describe COVID-19 accumulation curves (19, 20). This model has three parameters that correspond to the physical values of the phenomenon being described. Based on the model, predictions can be made that allow for obtaining a value for the maximum dose and enable the calculation of the time it will take this value. The modified Gompertz Model is presented by Equation 1.

(1) $$\text{ym}\left(\text{t}\right)\;={\text{ am}}^{.}\text{exp }\left[-\text{exp }\left({\left({\text{br}}^{.}\text{e}/\text{am}\right)}^{.}\left(\text{ct }-\text{ t}\right)\;+\;1\right)\right]$$

Where:

ym(t) – doses accumulation [drugs unit].

am – maximum doses [drugs unit].

br – doses rate [drugs unit/year].

ct – time of adaptation [year].

t – time [year].

e - basis of the natural logarithm [2.718].

**Figure 22:**
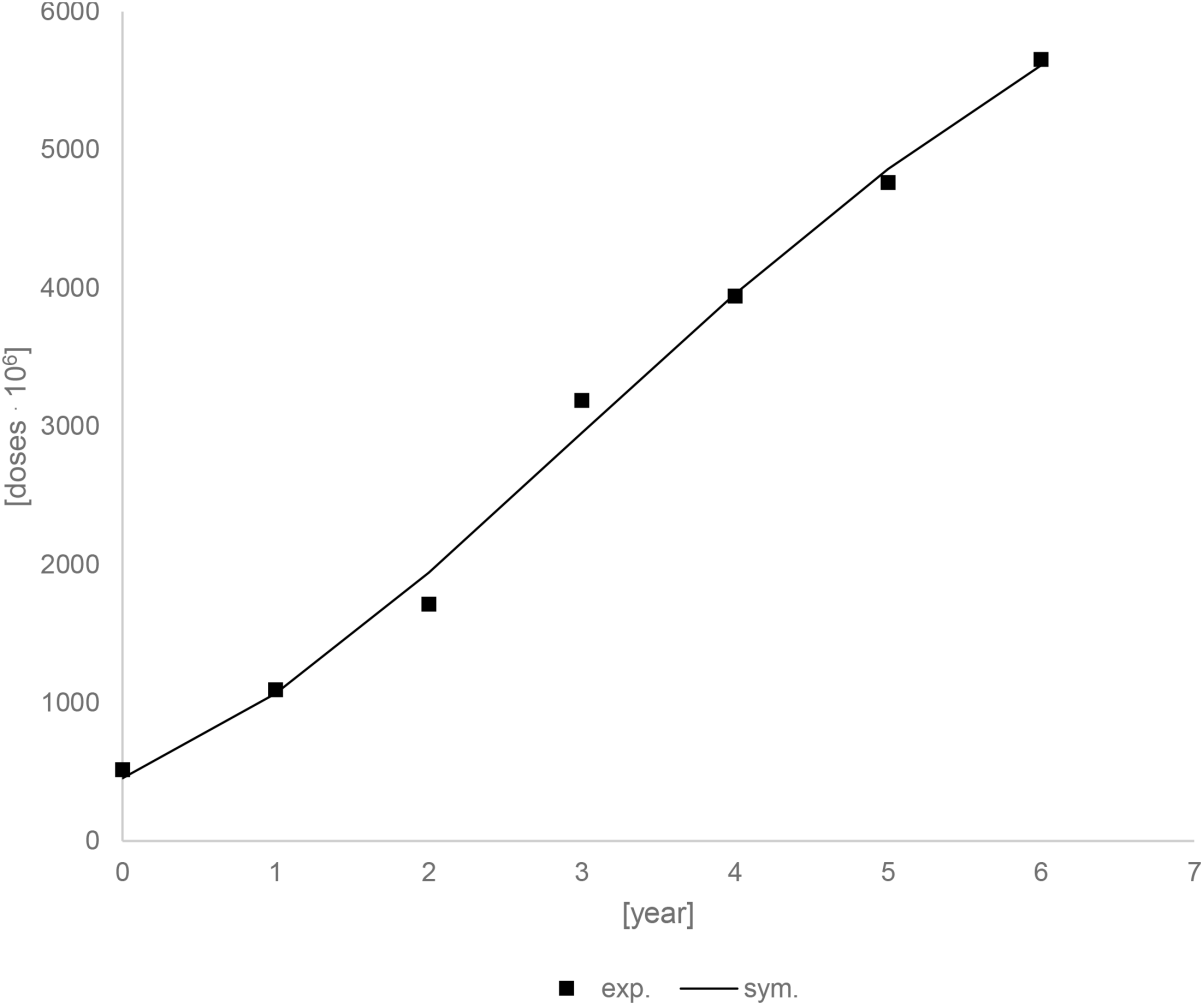
Experimental and simulation data for the drugs unit accumulation curve.

**Figure 22** shows the experimental and simulation data for the dose accumulation curve. The fit of the experimental results to the model results is good (R^2^ = 0,9946). The following values were obtained for the parameters: am_1_ = 7843,79.10^6^ [drugs unit], br_1_ = 1029,69.10^6^ [drugs unit/year], ct_1_ = 0,13 [year].

**Figure 23:**
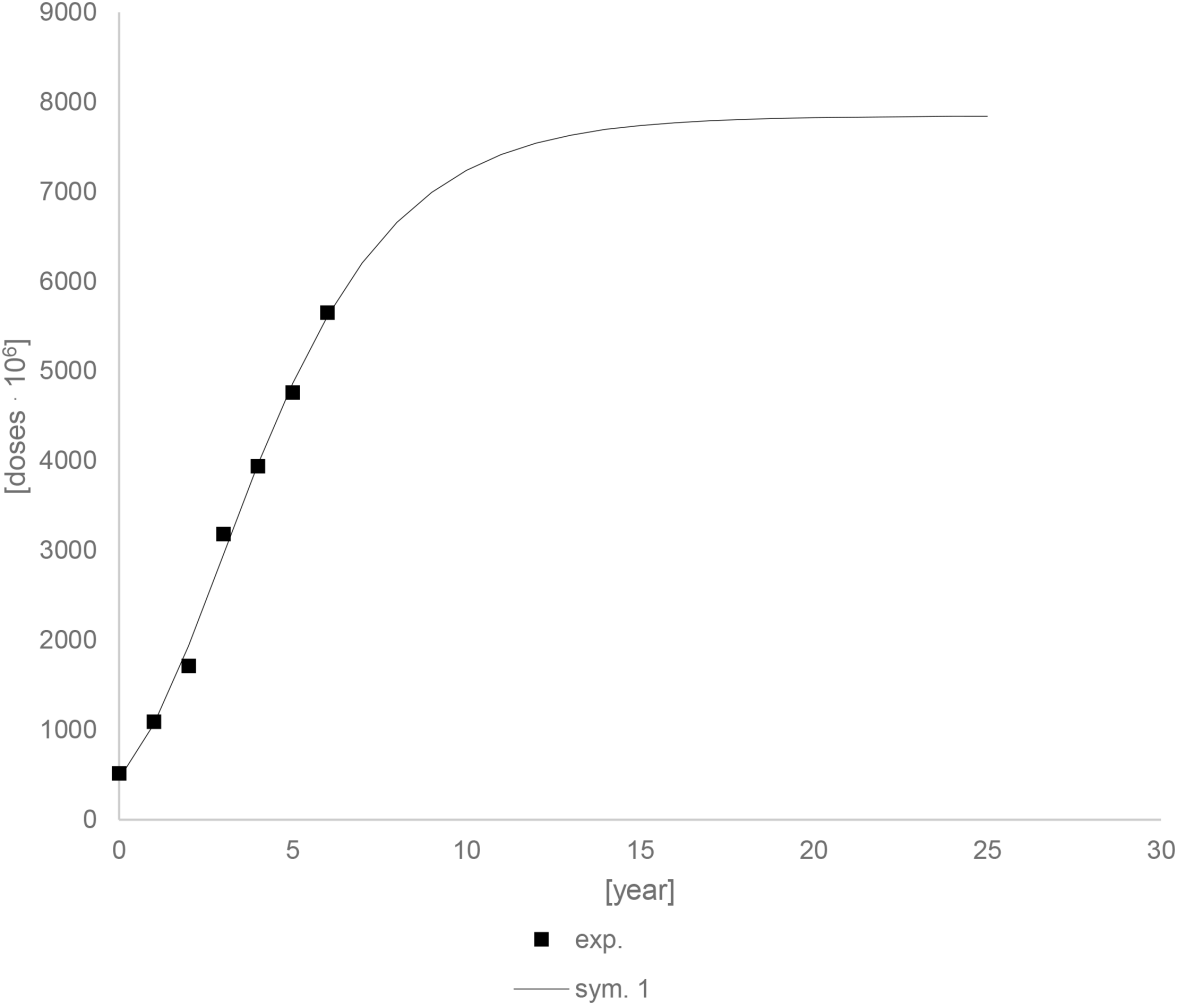
Experimental and simulation data for the drugs unit accumulation curve – prediction up to 25 years.

**Figure 23** shows the prediction up to 25 years using modeling data. Drug units should continue to grow for about 10 years, after which they should stabilize. After 10 years, there should be an increase of about 2877,70.10^6^ drugs units. This value is calculated for a situation in which no other factors occur during this time, which is not the case, but it can provide information about the trend.

**Figure 24:**
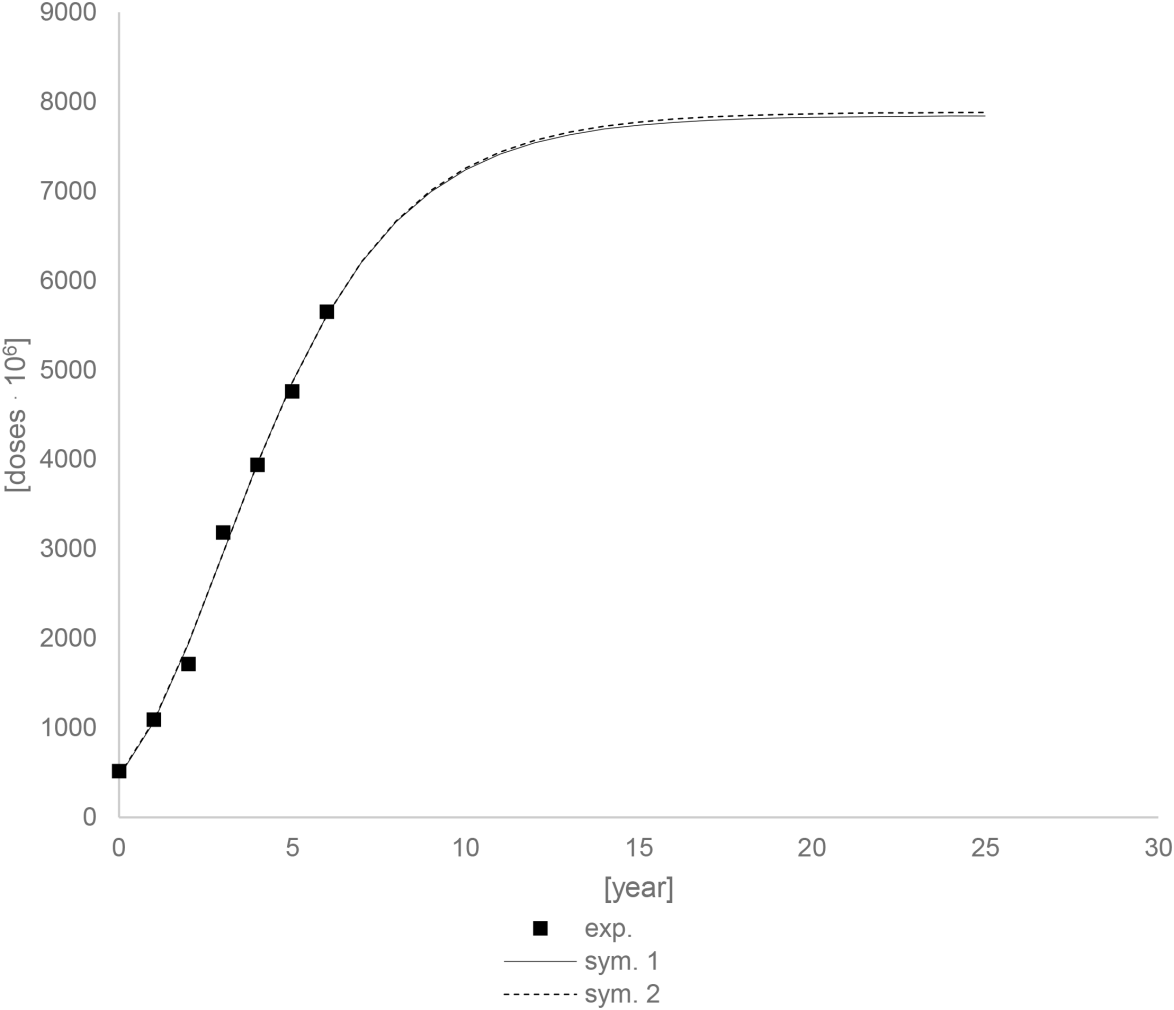
Comparison of simulations, sym. 1 and sym. 2 for different parameters.

Due to the uncertainty of the 2019 data, additional calculations were performed, which included determining constant parameters and conducting simulations with them. The following values were obtained for the parameters: am_2_ = 7883,84.10^6^ [drugs unit], br_2_ = 1025,39.10^6^ [drugs unit/year], ct_2_ = 0,12 [year], R^2^ = 0,9945. **Figure 24** shows comparison of simulations between different parameters. The simulation curves are almost identical.

## 4 Discussion

The COVID-19 pandemic has had a significant impact on the mental health of children and adolescents, as evidenced by numerous studies conducted in Poland. Research by Grzejszczak et al. demonstrated a substantial increase in depressive and anxiety symptoms among individuals under the age of 18 during the pandemic, as well as a rise in suicidal ideation and self-injurious behaviors, particularly among adolescents (21). Similar trends were observed in the study by Badzińska et al., where adolescents aged 10–15 from Kraków reported a decline in both mental and physical well-being, more frequent experiences of school-related stress, and increased fatigue (22).

Throughout the pre-COVID-19 period, the level of psychotropic drug dispensations in Polish children was stable, indicating the underlying rate of anxiety and depressive disorders and moderate tendency to prescribe medications in pediatric populations. While selective serotonin reuptake inhibitors (SSRIs) and serotonin-norepinephrine reuptake inhibitors (SNRIs) were already known as first-line treatments for pediatric depression and anxiety, their use was attenuated by regular in-person evaluations, limited subspecialty resources, and prescribing recommendations that favored psychosocial treatments over pharmacotherapy as first-line treatment (23, 24).

International evidence consistently shows that the utilization of child and adolescent mental health services is strongly shaped by socioeconomic gradients, residential context and system-level access factors, rather than by clinical need alone (24). Children from more affluent households, with higher parental education, and living in urban areas display higher rates of formalized help-seeking and treatment uptake, whereas unmet need and reliance on informal or non-specialist pathways are more prevalent in lower-SES and rural settings (25). In addition, the pandemic introduced further contextual heterogeneity: school closures and remote schooling altered detection opportunities and referral flows, regional variation in COVID-19 incidence influenced care avoidance behaviors, and access to private sector mental health services increased in major metropolitan areas when public-sector availability became constrained (26). Given that the current administrative aggregates do not allow stratification across these contextual domains, it is not possible to disentangle epidemiological change in symptom burden from shifts in access, care-seeking, diagnostic probability or the elasticity of substitution between public and private pathways. Therefore, the trends shown here should be interpreted as population-level utilization signals emerging from a dynamic socio-structural environment, rather than as direct proxies of changes in true underlying morbidity.

In early 2020, when the pandemic hit, that balance was turned upside down by an extraordinary convergence of stressors. In the context of school closure and social distancing, children had high levels of anxiety and depressive symptoms, but public-health campaigns and increased parental surveillance resulted in a higher rate of screening in primary-care and school settings. Importantly, the widespread adoption of telemedicine in Poland, where primary care teleconsultations increased substantially in 2020–2021, removed many of the usual obstacles to contacting psychiatric services and enabled clinicians to introduce and maintain intervention without face-to-face contact (27–29).

Similar trends have been observed in the United States, where a study conducted by the University of Michigan found that, since March 2020, the rate of antidepressant prescriptions among adolescents increased by 63.5%, with the highest rise noted among girls aged 12–17 an increase of 129.6% (30). The authors suggest that, in addition to the worsening of mental health, this increase may also be attributed to limited access to psychotherapy, which led physicians to rely more frequently on pharmacotherapy as a form of treatment. An increase in antidepressant prescriptions among adolescents was also recorded in the United Kingdom, particularly during lockdown periods, highlighting the global nature of this phenomenon (31).

Cross-national evidence further contextualizes these findings. In countries with well-developed community-based and preventive child mental health systems, such as the Nordic region and the Netherlands, pandemic-related increases in antidepressant prescribing among young people were modest and largely transient, suggesting that strong psychosocial infrastructures can buffer against long-term pharmacotherapy escalation (27, 28). In contrast, data from the United States showed a much steeper and more sustained rise in antidepressant dispensing among adolescents 63.5% faster after March 2020 compared with pre-pandemic trends reflecting a more medicalized response to youth mental health needs (29). Poland, with its historically hospital-centered and under-resourced system of child and adolescent psychiatry, appears to follow the latter trajectory. These contrasts highlight how system organization, access to non-pharmacological interventions, and the balance between medical and psychosocial resources shape prescribing dynamics as much as underlying epidemiological change.

Following the strictest restrictions lifted in 2022, dispensing of short-acting anxiolytics (benzodiazepines) decreased substantially, to or even below pre-pandemic levels, in line with population-level findings reporting an immediate drop in the use of pediatric benzodiazepine during and after the public-health emergency (32).

On the other hand, use of escitalopram, fluoxetine, venlafaxine, trazodone and quetiapine remained elevated until 2024, suggesting a chronic or recurrent course of depressive disorders or aggravated by the pandemic, according to multinational studies revealing the small magnitude but the sustainability of an antidepressant increase among children and adolescents in the later phases of COVID-19 (27, 33). A continuous (without a clear peak) increase in the use of sertraline was observed. This may be due to the fact that sertraline is considered a well-studied drug with a good safety profile and is therefore very often chosen, including for off-label treatment.

Data from the Social Insurance Institution (ZUS) in Poland indicate a growing number of sick leave days due to mental disorders among children and adolescents, which may reflect an increasing burden on the healthcare system and underscore the need to strengthen both preventive and intervention measures. In the international context, school principals in England report that mental health issues and anxiety are among the leading causes of increased school absenteeism following the COVID-19 pandemic, further confirming the global nature of the problem (29).

Recent studies emphasize that beyond pharmacological and system-level interventions, fostering health-promoting behaviors and active lifestyles plays a crucial role in mitigating pandemic-related psychological distress and supporting long-term mental well-being. Evidence from diverse populations, including Generation Z youth, older adults, and athletes demonstrates that engagement in physical activity, structured routines, and positive lifestyle habits can buffer the effects of stress, anxiety, and social disruption associated with COVID-19 (34–36). Integrating such behavioral components into national mental-health strategies for children and adolescents could enhance resilience and complement medical and psychotherapeutic interventions.

Both the increase in the use of pharmacotherapy and the number of sick leaves due to mental disorders among children and adolescents during the COVID-19 pandemic highlight the need to strengthen healthcare systems, improve access to psychotherapeutic services, and implement preventive and intervention programs. Further research is essential to understand the long-term effects of the pandemic on young people’s mental health and to develop effective support strategies. Together, these findings suggest that while acute situational anxiety diminished with the easing of social restrictions resulting in a reduced need for short-term anxiolytics, the impact of the pandemic on depressive disorders has been more persistent.

This underscores the importance of robust early intervention services and the development of comprehensive care models that integrate telepsychiatry with in-person psychiatric evaluations (34). Moreover, recent studies have demonstrated a sustained public interest in mental health issues during and after the pandemic, reflecting both increased awareness and growing unmet needs in this area (27, 37–40). These observations are consistent with clinical data indicating that the pandemic has contributed to enduring increases in depressive and insomnia symptoms across both psychiatric and general populations (41).

The exclusion of the lockdown months from the main regression models was a deliberate methodological choice intended to preserve analytic transparency and stability of the underlying trend rather than to overstate effects. The lockdown period constituted an exceptional disruption to healthcare provision marked by temporary suspension of in-person consultations, irregular prescription renewals, and system-level instability that violated the assumption of structural continuity required for time-series analysis. Including this anomalous period substantially reduced model fit and introduced volatility unrelated to typical prescribing behavior. By focusing on pre- and post-lockdown data, the analysis better reflects stabilized clinical and organizational conditions, allowing clearer interpretation of medium-term trajectories in pharmacotherapy. Nevertheless, supplementary checks confirmed that the overall direction of post-pandemic trends remained consistent when lockdown months were retained, suggesting that the exclusion improved interpretability without materially affecting substantive conclusions.

While the observed increase in psychotropic prescriptions among Polish children and adolescents clearly coincided with the COVID-19 pandemic, these findings should be interpreted with caution. The higher dispensing rates do not necessarily indicate a proportional rise in the true incidence or severity of depressive and anxiety disorders. Rather, they likely reflect a complex interplay of clinical, organizational, and social factors that evolved during the pandemic. Changes in prescribing practices and access to care may have played a significant role. The widespread adoption of tele-health and the temporary reduction in psychotherapy availability could have shifted the balance toward pharmacological management as a more accessible or expedient treatment option. Similarly, physicians’ greater vigilance in detecting emotional and behavioral symptoms, combined with parental concern, may have increased treatment-seeking behaviors and the number of prescriptions issued. Importantly, not all analyzed medications are prescribed for the same clinical indications. Several agents, particularly trazodone, hydroxyzine, and low-dose quetiapine, are frequently used off-label in pediatric populations to address insomnia, agitation, or behavioral dysregulation rather than primary mood or anxiety disorders (23, 27). This suggests that part of the observed rise may represent symptomatic or adjunctive treatment rather than the pharmacotherapy of clinically diagnosed depressive or anxiety syndromes. Accordingly, the interpretation of prescription trends as a direct proxy for mental disorder burden should be made with prudence. Future pharmacoepidemiological analyses should aim to differentiate drug utilization by indication, diagnostic category, and age subgroup, which would allow a more precise understanding of the mechanisms driving psychotropic medication use among Polish youth during and after the pandemic.

The presented results should also be viewed in the context of Poland’s aging population. For many years, the number of children (0–17 years of age) in Poland has been gradually decreasing. According to estimates by the Central Statistical Office (GUS- Główny Urząd Statystyczny), at the end of 2024, the size of this population was estimated at less than 6.8 million, which was about 100,000 fewer than at the end of 2023, and also much lower than a few decades earlier (compared to 2000, it decreased by over 2.5 million). This group in the year 2024 accounted for about 18% of the total Polish population, which is much less than in year 2000 (24.4%) (42).

Beyond the observed trends, the increase in pharmacotherapy among children and adolescents should be considered in light of potential risks and the need for balanced care. Psychotropic medications, while often necessary, may carry significant side effects and the risk of inappropriate or prolonged use without adequate monitoring. This underscores the importance of integrating pharmacological management with psychosocial and psychotherapeutic interventions as part of comprehensive, stepped care models recommended by international guidelines (NICE, CADDRA, WHO). Future studies should examine whether increased prescribing translates into improved clinical or functional outcomes, or whether it primarily reflects changes in access and treatment practices during and after the pandemic.

### 4.1 Significance of the study and practical implications

The present study is the first in Poland to examine such an extensive time span (2018–2024) in analyzing trends in psychotropic medication prescriptions, the number of sick leave days due to mental disorders, and the number of children and adolescents receiving psychiatric care in the context of the COVID-19 pandemic. Previous analyses have primarily focused on qualitative aspects or localized data, whereas this study is based on nationwide data and incorporates several key epidemiological indicators.

The findings have important implications for shaping future health policy and planning mental health care resources in Poland. They highlight the need to:

- expand access to psychiatric and psychotherapeutic services for children and adolescents,
- maintain and further develop telepsychiatry as a complement to in-person care,
- ensure early identification and effective treatment of mental disorders, particularly depression and anxiety,
- and strengthen preventive and educational programs within school and family environments.

In light of the increasing number of sick leave days related to children’s mental health and the sustained high level of pharmacotherapy following the pandemic, this study provides essential data for policymakers, clinicians, and health system planners. It may also serve as a reference point for future comparative research in other countries and as a foundation for evaluating the effectiveness of implemented reforms and support programs.

### 4.2 Study limitations

Despite the broad time frame and nationwide scope of the analyses, this study has several important limitations that should be taken into account when interpreting the results:

1. Aggregate and non-individual data – The analysis was based exclusively on aggregated administrative datasets (IQVIA sales data and ZUS national insurance statistics), which do not include individual-level clinical information such as patient age subgroups, comorbidities, illness severity, adherence, or the use of concurrent non-pharmacological interventions. As a result, the study cannot provide detailed insights into treatment appropriateness, therapeutic response, or patient-level predictors of psychotropic use. Moreover, the reliance on dispensed medication packages as a proxy for actual consumption limits the ability to infer adherence or real-world effectiveness, as prescriptions may not be filled, used consistently, or used for their primary indication. Future pharmacoepidemiological studies should aim to integrate prescription records with diagnostic, clinical outcome, and adherence data to enable a more comprehensive understanding of psychotropic medication use and its determinants in children and adolescents.
2. Lack of data on actual medication intake – The study analyzed data on dispensed medication packages, which does not necessarily reflect actual medication use by patients. Therefore, irregular intake, treatment discontinuation, or unfilled prescriptions cannot be ruled out.
3. Potential distortions due to changes in reporting and service availability – The sharp reduction in the number of patients observed in 2019 likely reflects a structural reporting discontinuity associated with the introduction of the new three-tier child and adolescent mental health care model in Poland (Level-I community psychological and psychotherapeutic centers were contracted and reported separately from classical child psychiatry units). To reduce the risk of biased inference, 2019 was explicitly treated as a structural break (binary indicator) in the primary longitudinal models, and sensitivity analyses excluding 2019 were performed; estimates of 2020–2024 trends remained directionally robust. At the same time, limited access to specialists during the pandemic may have delayed diagnoses or redirected patients to alternative forms of care (e.g., private services), which further complicates the interpretation of year-to-year fluctuations.
4. No direct information on symptom severity – ICD-10 codes do not fully capture the clinical profile of patients nor allow for the assessment of disorder severity. Consequently, it is difficult to draw conclusions about changes in the clinical characteristics of patients during the study period.
5. Contextual factors not included in the analysis – The study did not account for environmental and social variables (e.g., socioeconomic status, family situation, quality of school relationships), which may significantly influence the occurrence and course of mental disorders in children and adolescents. Moreover, the absence of stratification by socioeconomic status or urban versus rural residence means that we cannot distinguish whether the observed temporal variation primarily reflects true changes in morbidity or instead differential access dynamics across sociodemographic strata.
6. Lack of clinical outcome measures – The study relied on administrative and dispensing data and therefore cannot assess clinical outcomes such as symptom improvement, functional status, school performance, or quality of life. Consequently, it remains unclear whether increased psychotropic prescribing resulted in measurable benefits or adverse effects for children and adolescents. Future research combining registry data with clinical assessments or hospitalization records would provide a more complete understanding of treatment outcomes.
7. Modeling choices and generalizability – The exclusion of the lockdown period from the main regression models, although intended to preserve the interpretive clarity and stability of long-term trends, limits the generalizability of findings to this highly atypical phase. Sensitivity checks including lockdown months confirmed consistent directional patterns but with attenuated magnitudes, suggesting that the decision improved model interpretability without materially biasing results. In addition, the modified Gompertz projections presented in the Supplement are exploratory and should not be interpreted as forecasts, as they do not account for potential future changes in policy, clinical practice, or social context.

## 5 Conclusions

Three-time related phases of psychotropic prescription for Polish children are becoming evident. In the pre–COVID 19 era, yearly package counts were relatively consistent across drug classes. Every class, which includes SSRIs, SNRIs, anxiolytics and antipsychotics had a notable peak in dispensing in the pandemic year of 2021. In the years following the pandemic, use of benzodiazepines and related anxiolytics either returned to or fell below pre-pandemic levels, while antidepressant (particularly escitalopram, venlafaxine and trazodone) and antipsychotic (quetiapine) prescribing remained well above their pre-COVID levels. The results demonstrate an early spike in short-term anxiety treatment and a persisting higher level of depressive disorder medication after the COVID-19 upheaval.

The sick-leave day data indicates that the growth in mental-health-related sick-leave days increased abruptly over five years and took especially large leaps surrounding the arrival of the COVID-19 pandemic. This shift underlines the pressing need for increased preventive measures, including stress-management training and workplace wellness programs, and increased access to psychiatric and psychological care with the use of telemedicine and more specialists. Systems monitoring the well-being of workers would allow earlier recognition of risk and earlier targeted intervention, perhaps preventing further escalation of absence and reducing the social and economic costs.

As for the number of patients - the massive drop from 2018 to 2019 probably has something to do with reporting changes or program eligibility, not raw demand. The gradual increase was observed until 2022; there was an accelerated growth after 2022 until 2024, providing an indication of post-event accessibility fade-ins (eg, pandemic restrictions) and increased demand for child and adolescent mental-health services respectively. Service planners need to explore the reporting artefacts between 2018 and 2019, ensure capacity growth matches rising caseloads, particularly for depressive and anxiety disorders, and expand early-intervention programs to meet the increasing demand from this vulnerable age group.

## Data Availability

The original contributions presented in the study are included in the article/supplementary material. Further inquiries can be directed to the corresponding author.
